# The efficacy and safety of patent Foramen Ovale Closure for Refractory Epilepsy (PFOC-RE): a prospectively randomized control trial of an innovative surgical therapy for refractory epilepsy patients with PFO of high-grade right-to-left shunt

**DOI:** 10.1186/s12883-023-03317-0

**Published:** 2023-07-27

**Authors:** Ji Shuming, Li Hua, Tang Yusha, Chen Lei

**Affiliations:** 1 Department of Clinical Research Management, West China Hospital, Sichuan University, Chengdu, 610044 China; 2 Department of Neurology, West China Hospital, Joint Research Institution of Altitude Health, Sichuan University, Chengdu, 610044 China

**Keywords:** Patent foramen ovale closure, Refractory epilepsy, Seizure frequency control

## Abstract

**Background:**

A significant proportion of patients with epilepsy have an unknown etiology and lack effective targeted therapeutic drugs. Patent Foramen Ovale (PFO) induces hypoxia and microembolism, leading to cerebral neurological dysfunction and increased epilepsy risk. This study aims to assess the efficacy and safety of PFO closure for relieving epileptic seizures in patients with refractory epilepsy associated with PFO.

**Methods/design:**

Recruitment takes place at the West China Hospital of Sichuan University, China, for an open-label, randomized controlled clinical trial. The trial will include 110 patients with refractory epilepsy and PFO. Disease diagnoses will conform to the diagnostic criteria of the International League Against Epilepsy (ILAE) for refractory epilepsy and the American Society of Echocardiography (ASE) for PFO. Refractory epilepsy and high-grade right-to-left shunt (RLS) of the PFO will be further diagnosed using 24-hour video electroencephalogram and transthoracic echocardiography with contrast injection, respectively. Eligible participants require a secondary or higher volume of RLS.

**Trial registration:**

Chinese Clinical Trial Registry (ChiCTR2200065681). Registered on November 11, 2022.

**Supplementary Information:**

The online version contains supplementary material available at 10.1186/s12883-023-03317-0.

This trial will randomly assign participants to either the intervention or the control group. Participants in the intervention group will undergo PFO closure surgery and receive 24 weeks of antiplatelet therapy. Participants in the control group will only receive 24 weeks of antiplatelet therapy. The primary outcome of the trial is the percentage decrease in the frequency of epileptic seizures at 48 weeks postoperatively compared to that at preoperation. Secondary efficacy outcomes include percentage decrease in the average duration of seizures after operation, improvement in the severity of epilepsy, frequency of epileptiform discharge by 24-hour video electroencephalogram, postoperative quality of life evaluation, and improvement in migraine complications for participants who have them.

**Discussion**.

This trial is innovative, as it aims to show that PFO closure can benefit patients with refractory epilepsy and PFO by relieving epileptic symptoms and improving the quality of life while maintaining a high safety profile.

## Introduction

**Fig. 1:**
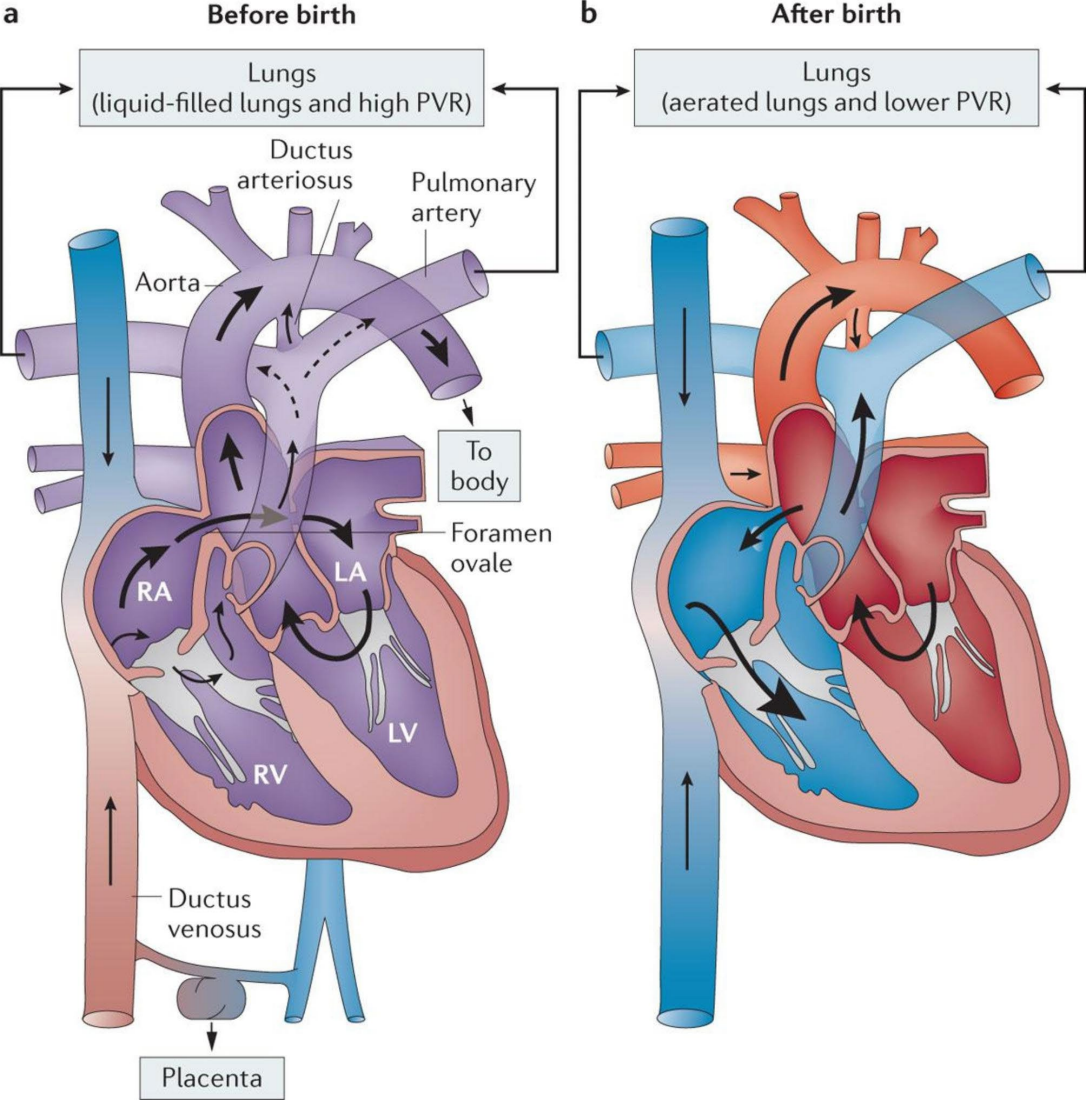
Schematic diagram of blood circulation in patients with patent foramen ovale before (Fig. 1a) and after birth (Fig. 1b). Note: PVR, pulmonary vascular resistance; RA, right atrium; LA, left atrium; RV, right ventricle; LV, left ventricle

Patent foramen ovale (PFO) is the most prevalent congenital heart abnormality of fetal origin, affecting approximately 25% of people worldwide [1]. Despite the lack of overt clinical symptoms, clinical evidences indicate that PFO is frequently found in patients with migraine, stroke, obstructive sleep apnea, and other central nervous system (CNS) diseases [2]. When addressing the causes of CNS diseases in individuals with PFO, a right-to-left shunt (RLS) (which is mediated by the PFO) is a significant factor in paradoxical embolization, which increases the risk of stroke and migraine [3]. Because the RLS is caused by PFO in the heart, some studies have shown that PFO may contribute to the process of microembolus-triggered cortical spreading depression [4], which is the primary explanation for the migraine aura induced by PFO [5]. In addition, the vasoactive substance hypothesis [6], high oxygen desaturation index/apne-hypopnea index [7], and associated biomarkers such as serum albumin-bound proteins [8] are few possible mechanisms that other researchers have proposed to explain the consequences of PFO in the brain. Fig. 1 shows a schematic of blood circulation in patients with PFO [2].

**Fig. 2:**
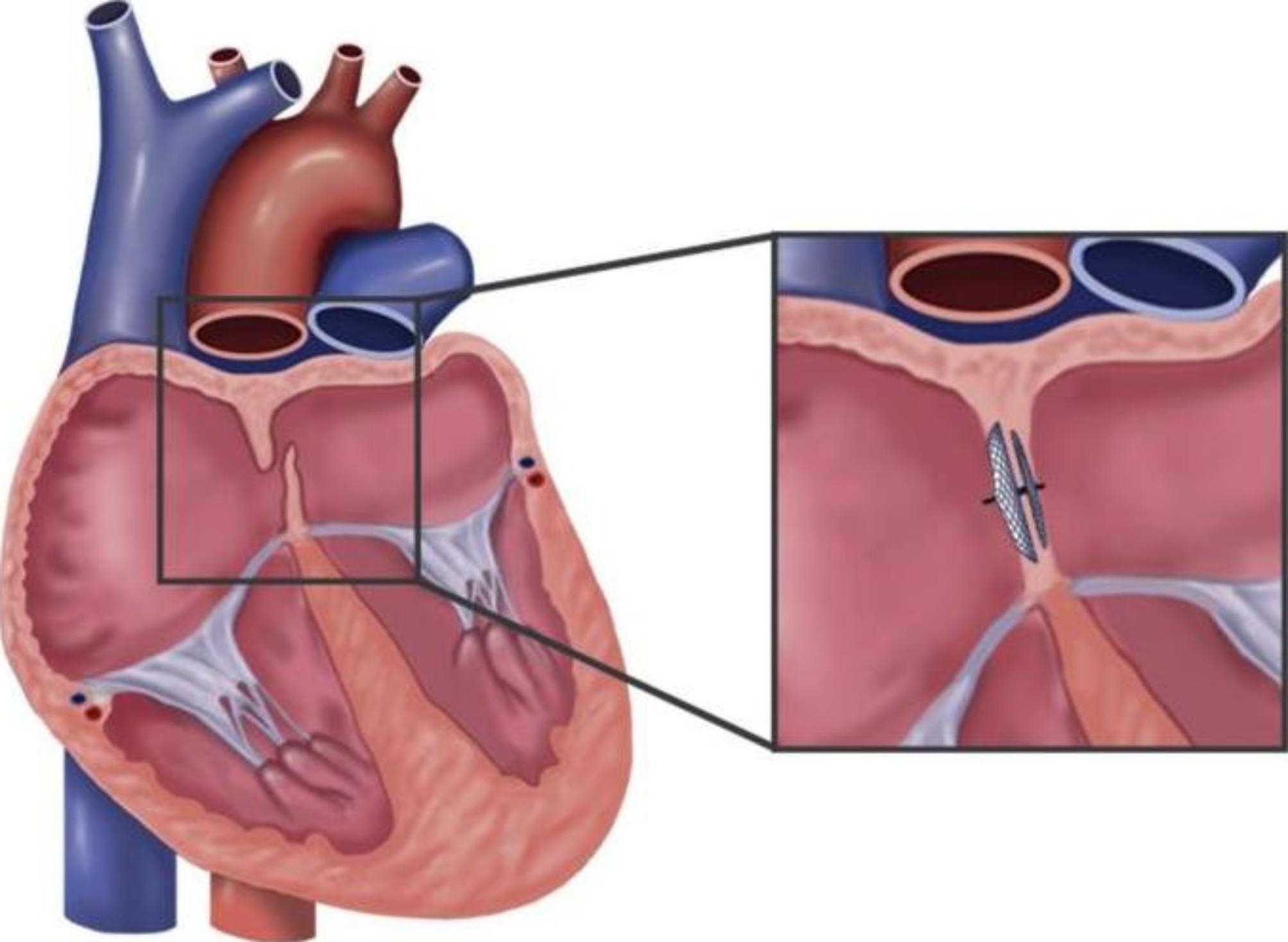
Schematic diagram of foramen ovale closure. Note: Local anaesthesia is first administered at the groin. The femoral vein is then punctured to facilitate venous access, followed by standard guide wire and catheter techniques to access the right side of the heart. Typically, closure of the patent foramen ovale (PFO) is accomplished by implantation of a self-expandable occluder under fluoroscopic as well as intracardiac echocardiography or transoesophageal echocardiography guidance. Periprocedural imaging of the PFO is not standard. The most widely used device to close the PFO is the double-disc-type occluder. The device catheter first crosses the PFO tunnel. The left-sided disk of the occluder is then completely unfolded and positioned at the left side of the interatrial septum. In a second step, the right-sided disk is unfolded and adopted to the right side of the septum. After verification of a secure and correct placement, the occluder is detached from the catheter and the remaining catheter material is retrieved. LA, left atrium; RA, right atrium

Prior to the introduction of percutaneous transcatheter closure, traditional surgical therapy was the primary method for PFO closure and was associated with low sealing effectiveness and numerous problems. The first case series documenting successful percutaneous transcatheter closure of PFO in 36 patients was published in 1992 [9]. Shortly thereafter, Schrader reported one of the largest series of PFO transcatheter closures involving 457 patients from 1994 to 2003 [10], including 169 patients with atrial septal aneurysms (ASA). These studies demonstrated the excellent safety and sealing efficacy of this procedure. Therefore, patients and their doctors are more likely to accept PFO closure using catheter-based treatments rather than surgical closure [11]. A Schematic diagram of foramen ovale closure is shown in Fig. 2 [12].

In recent years, PFO closure has demonstrated effectiveness and safety in individuals with certain CNS diseases, such as stroke and migraine, which co-occur with a high RLS grade of PFO. Long-term follow-up studies have shown that most patients who underwent PFO closure through catheter-based procedures were free from serious safety events and linked to a lower rate of recurrent ischemic strokes [13], a significant decrease in the average frequency of monthly migraine attacks [14], and a clear improvement in dyspnea and hypoxemia [15]. Therefore, the clinical benefits of PFO closure are increasingly attractive, and in 2022, evidence-based guidelines from the Society for Cardiovascular Angiography and Interventions provided appropriate patient selection and treatment recommendations for PFO closure in stroke and migraine [16].

Epilepsy affects over 69 million individuals worldwide and is one of the most common neurological diseases [17]. Unexpected and abrupt seizures in epilepsy lead to a high risk of disability and fatality and cause heavy mental pressure and disease burden for patients with epilepsy (PWE) [18]. Currently, approximately 50% of epilepsy cases do not have a definite cause, making it difficult to design efficient and targeted treatment [19]. Patients with epilepsy of unknown etiology have higher risks of seizures that are difficult to manage using medications [20]. Therefore, research on the potential unidentified causes of epilepsy is essential for accurate epilepsy treatment.

As one of the top ten CNS diseases, epilepsy is closely associated with dementia, migraine, and stroke [21]. For instance, PWE share several clinical characteristics with migraine, including episodic attacks, triggering factors, and the presence of aura [22]. In addition, they share some pathological mechanisms such as cortical spreading depression [23]. Therefore, considering the close connection between epilepsy, other CNS diseases, and the pathological mechanisms of PFO injury to the CNS, we hypothesized that PFO might be a possible cause of the development of refractory epilepsy, and PFO closure may contribute to relieving epileptic seizures.

To confirm this hypothesis, we did some preliminary work at the West China Hospital of Sichuan University, including screening PWE for the prevalence of PFO using contrast-enhanced transthoracic echocardiography, and observing the incidence of epilepsy in patients with PFO during long-term follow-up in a congenital heart disease cohort. We found that the PFO screening rate in PWE (38.69%) was higher than that in the general population (approximately 25%) [24], and the incidence of epilepsy in patients with PFO (4.61 per 1,000 person-years) was higher than that in the general population (approximately 0.61 per 1,000 person-years) [25]. Furthermore, we conducted PFO closure in 28 patients who were eligible for surgery, that is, patients with refractory epilepsy (PRE) accompanied by a second-grade or higher level of RLS, and found that both seizure frequency and severity were significantly reduced during two-year postoperative follow-up.

Based on this clinical evidence, we sought to perform a randomized clinical trial (RCT) using PFO closure to improve epileptic seizures in PRE accompanied by PFO of second-grade or higher RLS. We expect that this RCT would provide high-quality clinical evidence to verify our hypothesis and quantify the effectiveness and safety of PFO closure in these patients, laying the foundation for guiding clinical practice.

## Methods and design

### Study design and setting

This prospective, open-label, randomized, parallel-controlled clinical trial is conducted in the Epilepsy Center at the West China Hospital of Sichuan University. This study aims to evaluate the efficacy and safety of PFO closure for relieving epileptic seizures in patients with refractory epilepsy accompanied by PFO of second-grade or higher levels of RLS (PFOC-RE). The trial was registered on November 11, 2022, and the enrollment of all participants began on December 1, 2022. This trial is registered in the Chinese Clinical Trial Registry (ChiCTR2200065681).

**Fig. 3:**
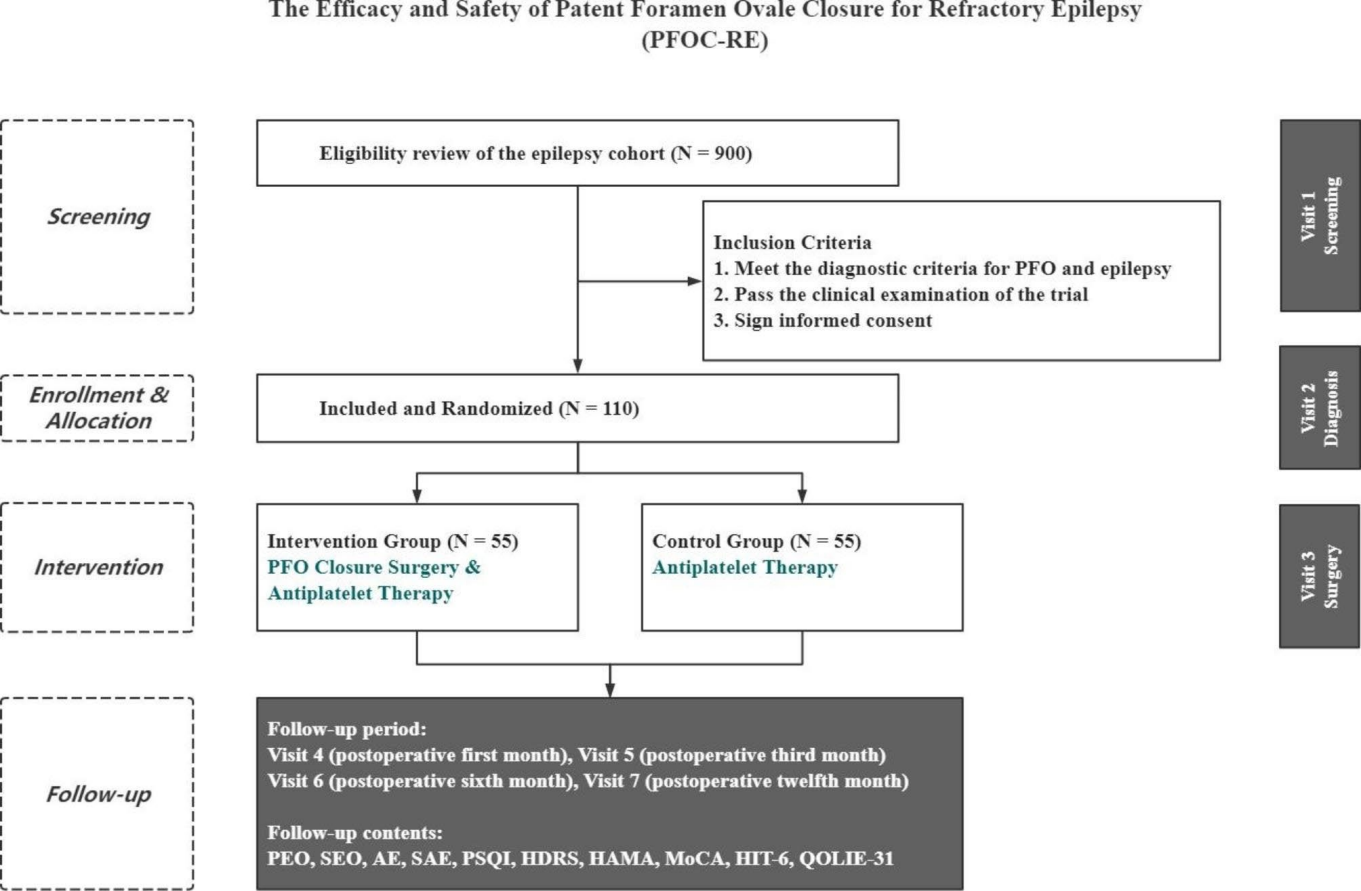
PFOC-RE Trial design flow chart. Note: PEO, primary efficacy outcome; SEO, secondary efficacy outcome; AE, adverse events; SAE, serious adverse events; PSQI, Pittsburgh sleep quality index scale; HDRS, Hamilton depression rating scale; HAMA, Hamilton anxiety scale; MoCA, Montreal cognitive assessment; HIT-6, Headache impact test; QOLIE-31, Quality of life in epilepsy inventory

After completing clinical screening and signing an informed consent form, all participants are randomized in a 1:1 ratio into the intervention or control group. Subjects assigned to the intervention group will undergo a PFO closure procedure and receive antiplatelet therapy for 24 weeks, while those in the control group will only receive antiplatelet therapy for 24 weeks, which is consistent with the medication regimen of the intervention group. All participants will be followed-up for 48 weeks. During the follow-up period, seizure characteristics including frequency, duration, severity, quality of life, and adverse safety events will be recorded to evaluate the efficacy and safety of the trial. The overall design flowchart is shown in Fig. 3. This trial adheres to the Standard Protocol Items: Consolidated Standards of Reporting Trials (CONSORT) Statement [26], which is outlined in **Supplement file 1**, and the trial registration details is found in **Supplement file 2**.

### Practitioners training

Research materials containing the information statement and research project protocol are formulated by the principal investigator. Participants are recruited according to a predetermined recruitment procedure and an informed consent form is handed over to each participant and saved. All cardiologists and neurologists involved in the trial studied the trial protocol and received program training from fieldworkers or through network conferences.

To facilitate the use of investigators and improve the cooperation of participants, researchers and research assistants will use electronic case report forms (eCRFs) and an online electronic data capture (EDC) system located in the hospital data center to collect and record the clinical information of the participants during the baseline and follow-up periods. Contributors to the data center will be responsible for data entry and standardization. Once the research is completed and the results are ready to be published, we shall share the identified data with the others.

### Participants eligibility

In this trial, we shall recruit all patients with refractory epilepsy accompanied by PFO of second-grade or higher levels of RLS from the epilepsy diagnosis and treatment center of West China Hospital of Sichuan University. Recruitment of participants began on December 1, 2022, and shall end on November 1, 2025. All participants will complete a series of clinical examination for epilepsy and PFO during the recruitment period, which lasts for approximately 6 weeks. Participants assigned into the intervention group will undergo the PFO closure one week after completing the recruitment period. The participants will be followed-up for 48 weeks after operation. Postoperative follow-up will end in November 30, 2026. A detailed trial schedule of the registration, interventions, and assessment is presented in Table 1, and a detailed description of the inclusion and exclusion criteria for the participants is presented in **Supplement file 3**.

**Table 1:** Schedule of enrollment, intervention, and assessment according to the Standard Protocol Items: Consolidated Standards of Reporting Trials (CONORT) statement

	Study Period								
	Screening	Enrollment	Allocation	Intra-operation	Post-operation				Close - out
Time Point*	-7 to -2	-1	-1	0	4	12	24	48	
**Screening**									
**General information**	**X**								
**Eligibility screening**	**X**								
**Epilepsy diary**	**X**								
**Enrollment**									
**Informed consent**		**X**							
**Allocation**			**X**						
**Interventions**									
**PFO closure**				**X**					
**Clopidogrel 75 mg**					**X**	**X**			
**Aspirin 100 mg**					**X**	**X**	**X**		
**Assessment**									
**Seizure frequency**					**X**	**X**	**X**	**X**	**X**
**Seizure duration**					**X**	**X**	**X**	**X**	**X**
**Seizure severity**					**X**	**X**	**X**	**X**	**X**
**Life quality**						**X**		**X**	**X**
**Headache score**						**X**		**X**	**X**
**Adverse events**					**X**	**X**	**X**	**X**	**X**

Note: * Times in weeks; PFO, Patent foramen ovale

### Inclusion criteria

**Fig. 4:**
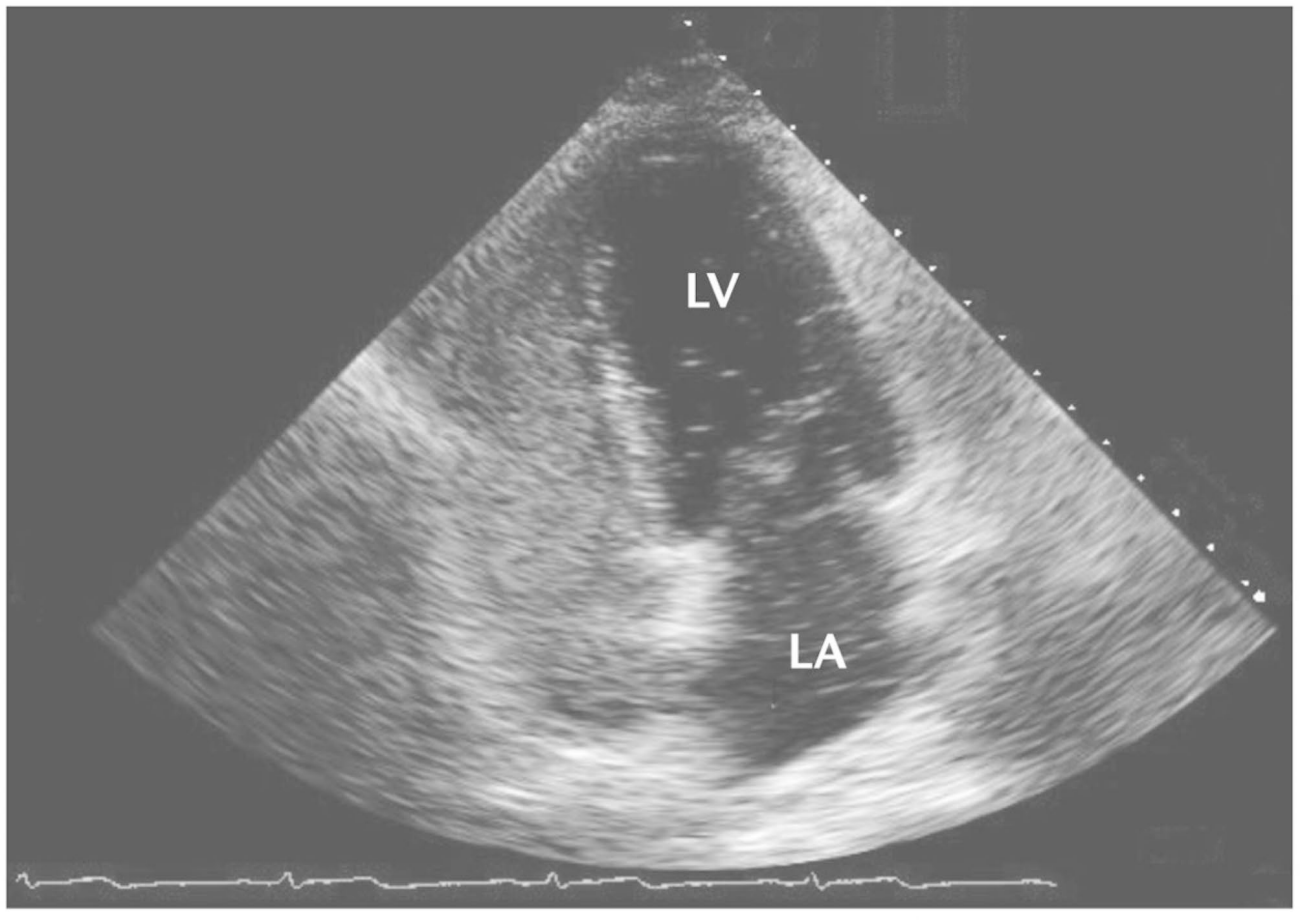
PFO detection by transthoracic echocardiography with contrast injection. Note: The right-sided cardiac chambers are completely opacified by contrast material (aerated saline) and shunting microbubbles are visualized in the left atrium (LA) and the left ventricle (LV). PFO, patent foramen ovale

1. It conforms to the diagnostic criteria for epilepsy by the International League Against Epilepsy, i.e., ILAE (2014 version).
2. It conforms to the diagnostic criteria for refractory epilepsy by the ILAE (2010 version).
3. It conforms to the diagnostic criteria for PFO by the American Society of Echocardiography (ASE) and Society for Cardiac Angiography and Intervention (SCAI) (2015 version). Participants are required to have a second or higher grade of RLS, detected using transthoracic echocardiography with contrast injection, as shown in Fig. 4 [2].
4. Patients aged 18 to 55 years-old, who can complete the epilepsy diary independently or with the help of family members.
5. Participants should have at least one epileptic seizure observed during the 6-week recruitment screening period and confirmed by a 24-hour video electroencephalogram (EEG) immediately after the seizure.
6. Participants are required to maintain a stable antiepileptic medical therapy during the screening period and throughout the trial, and to keep their medication regimen as unchanged as possible without an emergency event.
7. Participants were required to have a valid documented epilepsy diary for at least 4 weeks during the recruitment screening period.
8. Participants should agree to participate in this trial and sign an informed consent.

### Exclusion criteria

1. Participants with history of pseudo-seizure.
2. Participants with serious mental illness, such as anxiety or depression.
3. Participants with cognitive dysfunction (Mini-Mental State Examination score ≤ 23).
4. Participants with vascular puncture site infection or difficult puncture.
5. Participants with PFO associated with other cardiac structural abnormalities, such as moderate or higher valvular regurgitation and pulmonary hypertension.
6. Participants who have contraindications for antiplatelet therapy such as open trauma fracture surgery, gastrointestinal ulcers, active bleeding, and visceral bleeding three months before the screening period.
7. Participants with existing severe systemic diseases, such as digestive, circulatory, respiratory, liver, urinary, musculoskeletal, immune, or genetic metabolic diseases, whom researchers judge might be a potential impact on the results of the trial.
8. Participants who received head surgery or neuromodulation.
9. Participants under consideration for seizure-related surgery or any surgery involving general anesthesia, or who were already under general anesthesia four weeks before the screening period.
10. Participants who receive vaccinations during the screening period or four weeks before.
11. Participants preparing for pregnancy or breastfeeding during the trial period or three months after the study.
12. Patients who are participating in other interventional clinical studies during the trial.
13. Participants who are planning to travel or live abroad during the study period and cannot be followed-up.
14. Participants deemed inappropriate by the researchers for other reasons (need for detailed recording).

### Discontinuation criteria

Judgment according to the researchers.

1. Serious adverse events occur during the trial including severe cardiovascular and cerebrovascular emergencies, seizures with cardiac arrest, status epilepticus, or other symptoms of serious mental illness.
2. Adverse events occur during the trial, including allergies to antiplatelet drugs and intolerable side effects.
3. Participants who seriously violate the protocol.

Active withdrawal of the participants.

Reasons for active withdrawal from the trial include serious and common adverse events, loss to follow-up, and withdrawal of informed consent. Participants have the right to withdraw from the trial at any time and for any reason. To provide sufficient materials for the statistical analysis of this trial, when a participant decides to withdraw, the researchers will make efforts to complete the final examination, including recording follow-up information, arranging relevant clinical examinations, and documenting the eCRF. Detailed baseline information on participants and reasons for withdrawal shall be recorded in the eCRFs.

### Participant’s trial authority

In this trial, the participants are not involved in the design or discussion of the overall trial process, including the settings of the trial participants, interventions, controls, outcomes, and study design. The clinical examination and evaluation questionnaires are designed by a team of neurologists and cardiologists. In all other aspects, the participants will not be involved in the process of conduction or evaluation. However, a summary of the primary findings will be provided to all participating researchers and subjects for dissemination.

### Sample size

In our previous study, we conducted PFO closure in 28 cases of PRE accompanied by PFO in high-grade RLS and examined the seizure frequency of patients between one-year prior to surgery and one-year thereafter. We found that patients had an average decrease of 49.60% in seizure frequency one year after surgery compared to that before surgery, with a standard deviation of 41.67%.

Referring to the parameter settings in the studies on PFO closure in the treatment of cryptogenic stroke or migraine, the treatment regimen in the control group of this trial is consistent with that in the above studies. Therefore, we estimated that the average decrease in seizure frequency in the intervention group was 50%, with a standard deviation of 0.42; and an average decrease in the control group of 20%, with a standard deviation of 0.20. Combined with a superiority margin of 0.1, a single-sided alpha of 0.025, and a test power of 0.80, the total sample size required is 88. To account for possible participant withdrawal and loss to follow-up, we increase the sample size by 20%. Therefore, we shall recruit 110 subjects for this study [27].

### Randomisation and blinding

All participants will be informed of the purpose of the trial, sign an informed consent form, and complete a clinical examination at baseline. To balance the demographic characteristics between the trial groups, the participants will be randomized using block randomization with a variable block length. An independent statistician will use the R Project for Statistical Computing version 4.2.0 to perform the randomization procedure. The randomization sequence is generated using random block sizes of four and six in a 1:1 ratio. The allocation procedure is hidden, the randomized number is placed in opaque sealed envelopes and participants entering the trial will be randomly assigned to one of the envelopes and a grouping result based on the number. Blinding allocation cannot be masked by researchers and participants; however, the data analysts in our study will not be involved in any intervention and will remain blinded to the group allocation.

### Intervention and control

In the PFOC-RE trial, participants will be randomized to the intervention and control groups. The participants in the intervention group will undergo PFO closure and receive antiplatelet therapy for 24 weeks. Postoperative antiplatelet therapy involves two stages: a daily medication regimen with 75 mg of clopidogrel and 100 mg of aspirin from the first to the twelfth weeks, and a daily medication regimen with 100 mg of aspirin from the thirteenth to the twenty-fourth week. In contrast, the control group will only receive antiplatelet therapy for 24 weeks, which is consistent with the medication regimen in the intervention group.

### Follow-up procedure

All participants will begin the follow-up schedule on the first day of antiplatelet therapy. Participants will be interviewed at the end of the fourth, twelfth, twenty-fourth, and forty-eighth week. Four follow-up plans will be conducted, during which various examination parameters, seizure symptoms, PFO reviews, and mental health scale of postoperative recovery will be collected in detail for each participant.

### Data Collection and recording

During the recruitment and screening phases of the trial, baseline information, including demographic characteristics and relevant disease histories, will be collected. Epilepsy characteristics, seizure symptoms, medication records, a series of postoperative clinical examinations, and questionnaire surveys shall be conducted during the follow-up period.

#### Demographics

Participants‘ age, sex, height, weight, body mass index, smoking and drinking history, sleep pattern, date of birth, ethnicity, marital status, inpatient clinic card code, date of admission, trial number, telephone and mobile numbers, and addresses will be recorded.

#### Relevant diseases history

Participants will be asked about their history of extracorporeal circulation, congenital abnormalities, birth hypoxia, premature delivery, and febrile convulsions. They will also be asked if they have ever been diagnosed with a migraine, stroke, hypertension, or diabetes, and if they have a family history of epilepsy or associated comorbidities.

#### Epilepsy characteristics

Information on specific epilepsy characteristics, including the presence of aura, age of onset, seizure position, frequency and duration of seizures, assessment of seizure severity, and a detailed description of seizure symptoms will be collected from participants. In addition, researchers will gather information on the type and dosage of antiepileptic drugs used by the participants.

#### Clinical examination information

The participants will undergo a series of relevant clinical examinations during the follow-up period, including contrast-enhanced transesophageal echocardiography (cTTE), electrocardiography, 24-hour video electroencephalogram (EEG), head magnetic resonance imaging, routine blood examination, blood biochemistry, and urine analysis. In addition, they will be surveyed using several clinical scales, including the Hamilton’s Depression Scale, Hamilton’s Anxiety Scale, Mini-Mental State Examination, Montreal Cognitive Assessment Scale, Pittsburgh Sleep Quality Index Scale, Headache Impact Test Scale, and Quality of Life Scale.

### Study outcomes

#### Primary outcome

The primary efficacy outcome of this trial is the percentage decrease in the average seizure frequency per week during the postoperative 48-week compared to the preoperative period. Epileptic seizures must conform to the description of seizure characteristics in the ILAE, and the preoperative seizure frequency should be measured weekly and calculated based on valid seizure diaries only. The postoperative seizure frequency will be calculated in the same way, but should ensure that participants have valid seizure diaries for over 95% of the entire follow-up period.

### Secondary outcomes

Secondary efficacy outcomes include the percentage decrease in the average seizure duration per week during the postoperative 48 weeks compared to the preoperative period, improvement in postoperative epilepsy severity, frequency of epileptiform discharge by 24 h video EEG, evaluation of postoperative quality of life, improvement in migraine in patients with migraine complications, headache impact test, incidence rate of adverse events, cardiac ultrasound index, and laboratory indicators.

### Safety management and adverse events

The PFO closure surgery is a well-established interventional procedure with possible postoperative complications and serious adverse events, including (1) allergic reactions to anesthetics or contrast media. (2) High vagal response. (3) Major bleeding, hematoma, vascular dissection rupture, aneurysm, and arteriovenous fistula leading to mechanical hemolysis. (4) Severe arrhythmias such as atrial fibrillation. (5) Embolization. (6) Thrombocytopenia and severe platelet input. (7) Displacement or detachment of closure device material. (8) Catheter knotting, breakage, or injury. (9) Atrioventricular block. (10) Infection. 11. Hemothorax and pneumothorax. 12. Shock. 13. Cardiac tamponade. 14. Sudden death. 15. Heart failure. 16. Phrenic nerve palsy. 17. Arterial dissection, among others.

### Procedures of data management

A specialized team consisting of a statistician and neurologist will be responsible for data collection to ensure the confidentiality of information through electronic monitoring. This is done to ensure standardization of the study methods. The database was closely monitored for missing and redundant data. Logical checks and numerical range verifications were conducted automatically and manually. The data management processes include data entry, data submission, query generation, query replies by the corresponding researcher, feedback, and query resolution.

### Statistical analysis

#### Program of analysis set

The complete analysis plan consists of three sets: Full Analysis Set (FAS), Per-Protocol Analysis Set (PPS), and Safety Set (SS). The FAS and SS will include all randomly assigned participants, but those with serious protocol violations, active withdrawal, serious adverse events, or extremely poor compliance will be excluded from the PPS. For the FAS, we intend to follow the modified intention-to-treat (mITT) analysis principles, which exclude participants without a PFO diagnosis confirmed by intracardiac echocardiography during the surgical procedure.

### Missing data

If subjects are lost to follow-up during the study, their corresponding data will be carried over and included in the analysis. The project team’s doctors will evaluate the process, and the data will be reviewed by the data-monitoring committee.

### Statistical analysis methods

The demographic characteristics of PRE receiving PFO closure and those not receiving it will be compared to assess bias and ensure accurate representation of the participants. Primary and secondary outcomes will be described statistically using mean ± standard deviation, median, minimum, maximum, and other indicators. Categorical data will be described using frequencies and percentages.

The primary outcome of the trial will be analyzed using a mixed model of fixed and random effects, and factors with significant differences between the baseline datasets will be incorporated into the model as control variables. Additional analyses will include t-tests, chi-square tests, rank-sum tests, logistic regression, and propensity score matching to examine the secondary outcome indicators. Covariance analysis and mixed-effects models that consider the effect of time will be used to examine temporal trends in the clinical characteristics of epilepsy. All analyses will be performed using R-project version 4.2.1. Statistical significance will be set at two-sided p < 0.05.

## Discussion

The etiology of refractory epilepsy is complex and difficult to explore, and current drug therapies have not demonstrated satisfactory control efficacy. To the best of our knowledge, the PFOC-RE trial will be the first in the world to perform PFO closure in patients with refractory epilepsy accompanied by PFO of high-grade RLS and that covers at least a quarter of those with refractory epilepsy, which may bring encouraging news. This study aims to achieve good treatment effects in relieving epileptic symptoms in patients with refractory epilepsy and PFO, including reducing the frequency of postoperative seizures and alleviating seizure symptoms. However, there are still some limitations in this trial. Due to patients into the intervention group should undergo PFO closure, it is hard to effectively mask protocol for the patients and physicians, which may cause bias and may need to be verified in future real-world studies with larger samples.

The mechanism of the impact of the PFO on brain neurofunction has been gradually clarified, and there is enough clinical evidence of the health benefits of PFO closure in patients with cryptogenic stroke and migraines [13]. Our clinical trial may further expand the therapeutic value of PFO closure in neurofunctional brain diseases. Importantly, our study results provide clinical evidence for exploring the mechanism between PFO and refractory epilepsy and may be used in future larger multicenter clinical trials for the benefit of more patients.

### Trial status

#### Ethical approval

for this trial was received from the Ethics Committee of West China Hospital of Sichuan University (No. HXDZ21006). The first participant was enrolled on December 1, 2022. We expect to finalize the study by November 30, 2026. Protocol version 1.0 document completed in December 2022. Any modification to the protocol that may affect the conduct of the study, the potential benefit to patients, or patient safety must be approved by the Ethics Committee before implementation.

## Electronic supplementary material

Below is the link to the electronic supplementary material.

Supplementary Material 1

Supplementary Material 2

Supplementary Material 3

Supplementary Material 4

## Data Availability

The datasets generated during the current study are not publicly available because of the confidentiality requirements for patients’ personal and clinical information of the hospital but are available from the corresponding author upon reasonable request.

## Declarations

### Protocol amendments

Modifications to the trial procedures will be documented by protocol amendments.

### Protocol

Shown in **supplement file 2**.

## Abbreviations

Patent Foramen Ovale Closure for Refractory Epilepsy: PFOC-RE
Patent foramen ovale: PFO
Right-to-left shunt: RLS
Randomized controlled trial: RCT
Central nervous system: CNS
Atrial septal aneurysms: ASA
patients with epilepsy: PWE
Patients with refractory epilepsy: PRE
Consolidated Standards of Reporting Trials: CONSORT
Electronic case report forms: eCRFs
Electronic data capture: EDC
International League Against Epilepsy: ILAE
American Society of Echocardiography: ASE
Society for Cardiac Angiography and Intervention: SCAI
Mini-Mental State Examination: MMSE
Contrast-enhanced transesophageal echocardiography: cTTE
Electroencephalo-graph: EEG
Full analysis set: FAS
Per-protocol analysis set: PPS
Safety set: SS
Modified intention-to-treat: mITT
