# Supplementary material for: The efficacy and safety of patent Foramen Ovale Closure for Refractory Epilepsy (PFOC-RE): a prospectively randomized control trial of an innovative surgical therapy for refractory epilepsy patients with PFO of high-grade right-to-left shunt
Source: BMC Neurol. 2023 Jul 27;23:282. doi: 10.1186/s12883-023-03317-0 (PMC10373383; doi:10.1186/s12883-023-03317-0)
Supplement: Supplementary file 2 — Supplementary Material 2 [file 12883_2023_3317_MOESM2_ESM.docx]

**Protocol**

| **The Efficacy and Safety of Patent Foramen Ovale Closure for Relieving Epileptic Seizures in Patients**  **with Refractory Epilepsy (PFOC-RE)**  **A prospective randomized control trial of an innovative treatment procedure for refractory epilepsy patients with PFO of high-grade right-to-left shunt**  **THE OPEN STUDY**  **Chinese Clinical Trial Registry: ChiCTR2200065681**  **Version 1.0 Registration Date 11/11/2022** |
| --- |

Promoter: West China Hospital of Sichuan University

Principal Investigator: Lei Chen, Professor, Doctor of Neurology

Address: 37 Guoxue Lane, Wuhou District, Chengdu City, Sichuan Province

TEL: 086+189-8060-5819

E-mail: [leilei_25@126.com](mailto:leilei_25@126.com)

Funder:

Medical and Health Technology Innovation Project of Chinese Academy of Medical Sciences, No.2022-I2M-C&T-B-100.

1-3-5 project for disciplines of Excellence Clinical Research Incubation Project, West China Hospital, Sichuan University, No. 2021HXFH012.

**1. Protocol Summary**

Nearly 40% of epilepsy patients with an unknown etiology and lack effective targeted therapeutic drugs. PFO is known to induce hypoxia and micro-embolism, leading to cerebral neurological dysfunction and increased risk of epilepsy. PFO closure surgery has been introduced into the corresponding clinical guidelines, because of its significant effect in patients with cryptogenic stroke and migraine. The PFOC-RE trail aims to evaluate the efficacy of PFO closure surgery for refractory epilepsy patients, and assesses its potential clinical value of improving epileptic seizures in patients with PFO and epilepsy. Recruitment takes place at the West China Hospital of Sichuan University, China, with started in December 2022 until November 2026, for an open-label, randomized controlled clinical trial. This trial will includes 110 patients diagnosed with refractory epilepsy and PFO, which based on ILAE's diagnostic criteria for epilepsy and ASE's diagnostic criteria for PFO using echocardiography. Eligible patients with epilepsy require a middle (over than 10 microbubbles) or higher volume of right-to-left shunt. Patients will randomly assigned to either surgical intervention group or control group. Patients in intervention group will undergo PFO closure surgery and receive six months of antiplatelet therapy, and will only receive six months of antiplatelet therapy in control group. The primary outcome is the percentage of decline in the frequency of epileptic seizures during the first year after surgery compared to before surgery. Secondary efficacy outcomes include the percentage of decline in the average duration of seizures after surgery, alleviation in the severity of epilepsy, frequency of epileptiform discharge by video EEG, and postoperative quality of life. PFOC-RE is an innovative and clinically significant trail, which aims to identify whether PFO closure surgery can benefit patients with refractory epilepsy and PFO by relieving symptoms and improving quality of life while maintaining a high safety profile.

**2. Background**

Patent foramen ovale (PFO) is the most common congenital heart abnormality of fetal origin, and presents in approximately 25% of adults worldwide[1]. Although PFO has no obvious clinical symptoms, clinical evidences suggest that it is closely associated with migraine, stroke, obstructive sleep apnea and other neurological diseases[2]. In discussing the mechanisms of neurological diseases in patients with PFO, an important viewpoint is that the presence of paradoxical embolization increases the risk of stroke and migraine, and the right-to-left shunt (RLS) mediated by PFO is an important cause of paradoxical embolization[3]. Due to the existence of RLS in the heart, some studies suggest that PFO may play a role in the process of microembolus-triggered cortical spreading depression[4], which is the main theory explaining the aura of migraine[5]. Additionally, other research proposes alternative mechanisms, such as the vasoactive substance hypothesis[6], increased oxygen desaturation index / apne-hypopnea index[7], as well as the discovery of biomarkers, such as serum albumin-bound proteins, to assess the effects of PFO endovascular closure[8]. A blood circulation diagram for PFO patients is shown in Figure 1[2].


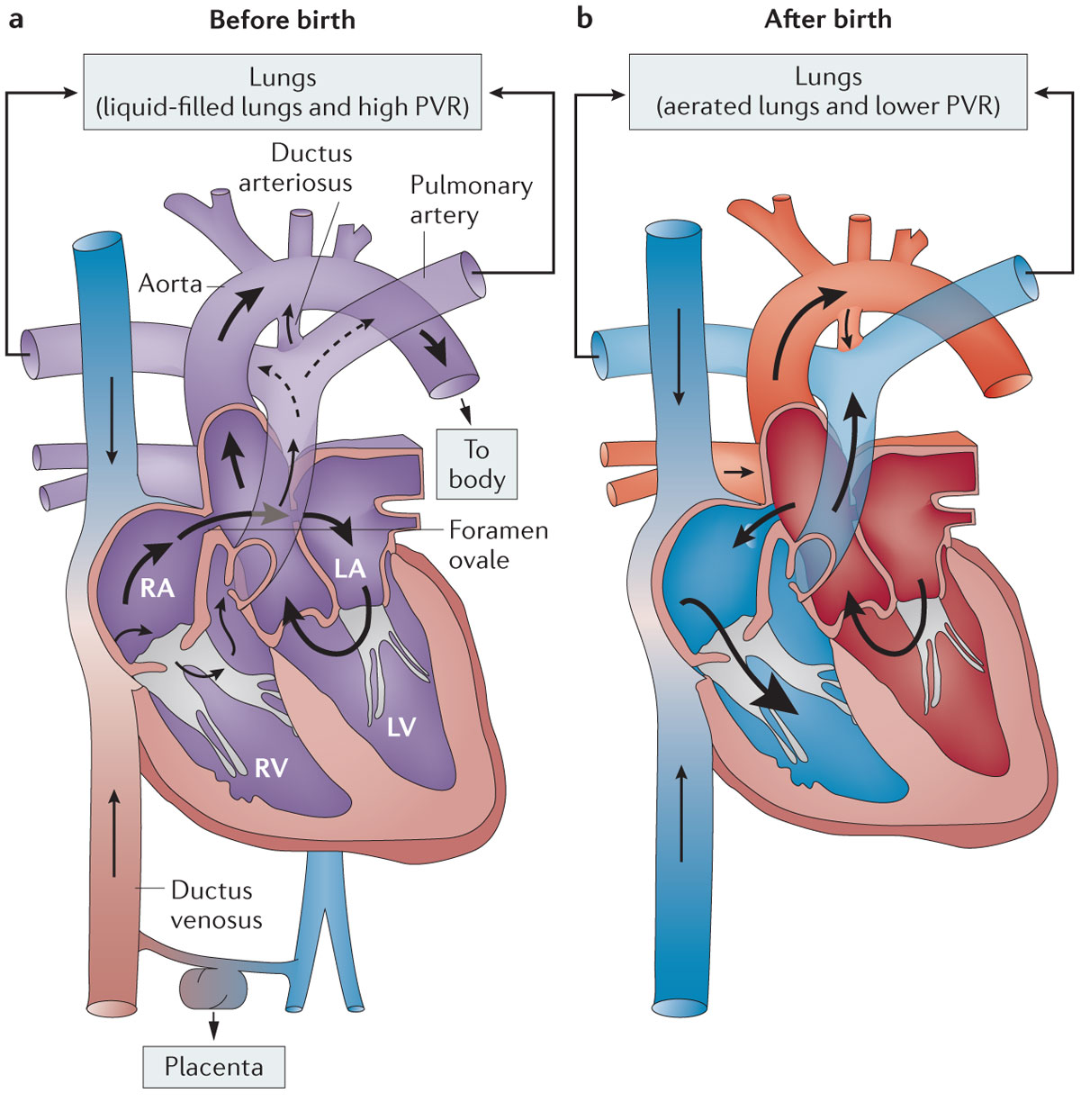


**Fig.1 Schematic diagram of blood circulation in patients with patent foramen ovale before and after birth.**

Note: PVR, pulmonary vascular resistance; RA, right atrium; LA, left atrium; RV, right ventricle; LV, left ventricle.

Surgical treatment has been the primary method of PFO closure all the time before the development of percutaneous trans-catheter closure, which was associated with poor sealing efficacy and various complications. The first case series reporting successful percutaneous trans-catheter closure of PFO in 36 patients was published in 1992[9], soon after, Schrader reported one of the largest series of PFO trans-catheter closures involving 457 patients, including 169 with atrial septal aneurysm (ASA), from 1994 to 2003[10]. The study demonstrated the great safety and sealing efficacy of this operation. Therefore, the PFO closure though the catheter-based procedures, instead of surgical closure, has become widely accepted among patients and their doctors[11].

The efficacy and safety of PFO closure in patients with stroke, migraine, and other nervous system diseases have been successively confirmed in recent years. Long-term follow-up studies have also shown that most patients who underwent PFO closure through catheter-based procedures not only without serious safety events, but also indeed associated with a lower rate of recurrent ischemic strokes[12], a significant reduction in the mean number of monthly migraine attacks[13], as well as an obvious improvement of dyspnea and hypoxemia[14]. In 2022, evidence-based guidelines from the Society for Cardiovascular Angiography and Interventions provided appropriate patient selection and treatment recommendations for PFO closure in stroke and migraine[15]. A Schematic diagram of foramen ovale closure shown in Figure 2[16].


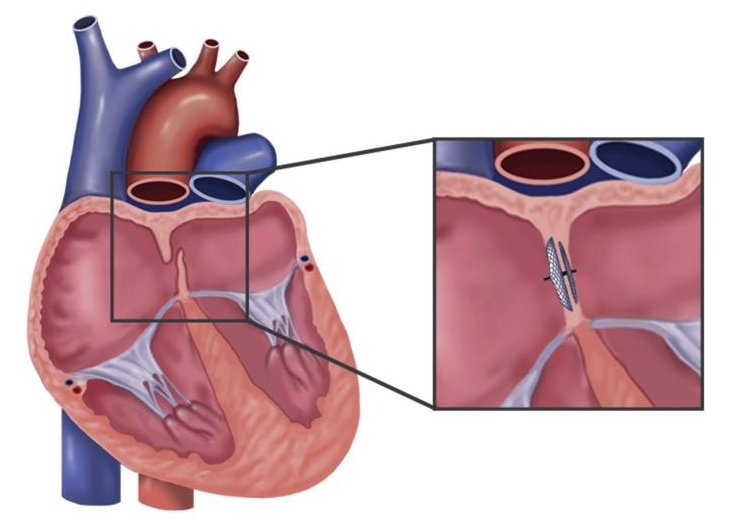


**Fig.2 Schematic diagram of foramen ovale closure.**

Note: Local anaesthesia is first administered at the groin. The femoral vein is then punctured to facilitate venous access, followed by standard guide wire and catheter techniques to access the right side of the heart. Typically, closure of the patent foramen ovale (PFO) is accomplished by implantation of a self-expandable occluder under fluoroscopic as well as intracardiac echocardiography or transoesophageal echocardiography guidance. Peri-procedural imaging of the PFO is not standard. The most widely used device to close the PFO is the double-disc-type occluder. The device catheter first crosses the PFO tunnel. The left-sided disk of the occluder is then completely unfolded and positioned at the left side of the interatrial septum. In a second step, the right-sided disk is unfolded and adopted to the right side of the septum. After verification of a secure and correct placement, the occluder is detached from the catheter and the remaining catheter material is retrieved. LA, left atrium; RA, right atrium

Epilepsy is one of the most prevalent neurological diseases, affecting approximately 69 million people worldwide[17]. The unpredictable and sudden seizures characteristic of epilepsy result in a high disability and mortality rate, and causing a severe mental pressure and disease burden on both patients and society as a whole[18]. Presently, nearly 50% of epilepsy cases have no discernible cause, making it challenging to develop effective targeted treatments[19]. This not only contributes to the development of refractory epilepsy but also leads to a high recurrence rate[20]. Therefore, investigating potential unknown etiologies of epilepsy is crucial for accurate treatment of this disease.

As a nervous system disease with a heavy disease burden, epilepsy is closely associated with several other nervous system diseases[21]. For instance, epilepsy shares several clinical features with migraine, such as episodic attacks, triggering factors, presence of aura, and frequent familiarity[22]. Additionally, they share several mechanisms such as cortical spreading depression[23]. PFO has been found to affect stroke and migraine through mechanisms such as paradoxical embolization, microembolus triggered cortical spreading depression, and low oxygen saturation. It is possible that PFO may also be a risk factor for refractory epilepsy patients by these mechanisms. Based on this, we propose the hypothesis that PFO is an independent risk factor for refractory epilepsy and that PFO closure may helpful to control seizures in these patients.

In our preliminary work, we screened epilepsy patients at the Epilepsy Center of West China Hospital at Sichuan University for patent foramen ovale (PFO) using contrast-enhanced transthoracic echocardiography. We found that the prevalence of PFO was much higher in epilepsy patients than in the general population (PFO prevalence: 38.69%). Additionally, we observed a higher incidence of epilepsy in the PFO cohort than in the normal population (Epilepsy incidence: 2.15%). Furthermore, we conducted surgical treatment on 28 refractory epilepsy patients with PFO, and found that both seizure frequency and severity decreased significantly during the 2-year postoperative follow-up period. Building on these research results, we conducted a clinical randomised trial to provide powerful evidence for the efficacy of PFO closure in refractory epilepsy patients with PFO.

**3. Reference**

1. Teshome, M.K., et al., *Patent Foramen Ovale: A Comprehensive Review.* Curr Probl Cardiol, 2020. **45**(2): p. 100392.

2. Homma, S., et al., *Patent foramen ovale.* Nat Rev Dis Primers, 2016. **2**: p. 15086.

3. Le Moigne, E., et al., *Patent Foramen Ovale and Ischemic Stroke in Patients With Pulmonary Embolism: A Prospective Cohort Study.* Ann Intern Med, 2019. **170**(11): p. 756-763.

4. Cao, W., et al., *The Patent Foramen Ovale and Migraine: Associated Mechanisms and Perspectives from MRI Evidence.* Brain sciences, 2022. **12**(7): p. 941.

5. Ashina, M., et al., *Migraine and the trigeminovascular system-40 years and counting.* Lancet Neurol, 2019. **18**(8): p. 795-804.

6. Wilmshurst, P. and S. Nightingale, *The role of cardiac and pulmonary pathology in migraine: a hypothesis.* Headache, 2006. **46**(3): p. 429-34.

7. Shaikh, Z.F., et al., *Patent foramen ovale in severe obstructive sleep apnea: clinical features and effects of closure.* Chest, 2013. **143**(1): p. 56-63.

8. Lopez, M.F., et al., *Proteomic signatures of serum albumin-bound proteins from stroke patients with and without endovascular closure of PFO are significantly different and suggest a novel mechanism for cholesterol efflux.* Clinical proteomics, 2015. **12**(1): p. 2-2.

9. Bridges, N.D., et al., *Transcatheter closure of patent foramen ovale after presumed paradoxical embolism.* Circulation, 1992. **86**(6): p. 1902-8.

10. Schräder, R., *Indication and techniques of transcatheter closure of patent foramen ovale.* J Interv Cardiol, 2003. **16**(6): p. 543-51.

11. Akagi, T., *Transcatheter closure of patent foramen ovale: Current evidence and future perspectives.* J Cardiol, 2021. **77**(1): p. 3-9.

12. Saver, J.L., et al., *Long-Term Outcomes of Patent Foramen Ovale Closure or Medical Therapy after Stroke.* New England Journal of Medicine, 2017. **377**(11): p. 1022-1032.

13. Mojadidi, M.K., et al., *Pooled Analysis of PFO Occluder Device Trials in Patients With PFO and Migraine.* J Am Coll Cardiol, 2021. **77**(6): p. 667-676.

14. Mojadidi, M.K., et al., *Patent Foramen Ovale and Hypoxemia.* Cardiol Rev, 2019. **27**(1): p. 34-40.

15. Kavinsky, C.J., et al., *SCAI Guidelines for the Management of Patent Foramen Ovale.* Journal of the Society for Cardiovascular Angiography & Interventions, 2022. **1**(4).

16. Wintzer-Wehekind, J., et al., *Long-Term Follow-Up After Closure of Patent Foramen Ovale in Patients With Cryptogenic Embolism.* J Am Coll Cardiol, 2019. **73**(3): p. 278-287.

17. Rawat, C., et al., *Cyclooxygenase-2 (COX-2) inhibitors: future therapeutic strategies for epilepsy management.* J Neuroinflammation, 2019. **16**(1): p. 197.

18. *Global, regional, and national burden of epilepsy, 1990-2016: a systematic analysis for the Global Burden of Disease Study 2016.* Lancet Neurol, 2019. **18**(4): p. 357-375.

19. Neligan, A., W.A. Hauser, and J.W. Sander, *The epidemiology of the epilepsies.* Handb Clin Neurol, 2012. **107**: p. 113-33.

20. Ryvlin, P., M. Cucherat, and S. Rheims, *Risk of sudden unexpected death in epilepsy in patients given adjunctive antiepileptic treatment for refractory seizures: a meta-analysis of placebo-controlled randomised trials.* Lancet Neurol, 2011. **10**(11): p. 961-8.

21. *Global, regional, and national burden of neurological disorders, 1990-2016: a systematic analysis for the Global Burden of Disease Study 2016.* Lancet Neurol, 2019. **18**(5): p. 459-480.

22. Nye, B.L. and V.M. Thadani, *Migraine and epilepsy: review of the literature.* Headache, 2015. **55**(3): p. 359-80.

23. Zarcone, D. and S. Corbetta, *Shared mechanisms of epilepsy, migraine and affective disorders.* Neurol Sci, 2017. **38**(Suppl 1): p. 73-76.

**4. Study purpose and design**

The PFOC-RE trial is a prospective, open-label, randomized, parallel-controlled study designed to investigates the efficacy and safety of PFO closure surgery for the treatment of refractory epilepsy patients, and PFOC-RE followed a 1:1 allocation ratio and was an superiority trail. The primary aim of the trial is to assesses the capacity of PFO closure surgery to alleviate epileptic seizures in patients with epilepsy and PFO.

**5. Methods: Participants, interventions and outcomes**

**5.1** **Study setting and procedure**

This prospective, open-label, randomized, parallel-controlled clinical trial is conducted in the Epilepsy Center at the West China Hospital of Sichuan University. This study aims to evaluate the efficacy and safety of PFO closure for relieving epileptic seizures in patients with refractory epilepsy accompanied by PFO of second-grade or higher levels of RLS (PFOC-RE). The trial was registered on November 11, 2022, and the enrollment of all participants began on December 1, 2022. This trial is registered in the Chinese Clinical Trial Registry (ChiCTR2200065681).

After completing clinical screening and signing an informed consent form, all participants are randomized in a 1:1 ratio into the intervention or control group. Subjects assigned to the intervention group will undergo a PFO closure procedure and receive antiplatelet therapy for 24 weeks, while those in the control group will only receive antiplatelet therapy for 24 weeks, which is consistent with the medication regimen of the intervention group. All participants will be followed-up for 48 weeks. During the follow-up period, seizure characteristics including frequency, duration, severity, quality of life, and adverse safety events will be recorded to evaluate the efficacy and safety of the trial. The overall design flow chart is shown in Figure 3, and the detailed study period is presented in Table 1.

**5.2 Participants Eligibility**

Participants included not only patients but also the staff of the research team. For patients, we set a series of inclusion and exclusion criteria, which could seen in Supplement file 3, and for practitioners, we also set recruitment conditions and training procedures.

Inclusion criteria

1.It conforms to the diagnostic criteria for epilepsy by the International League Against Epilepsy, i.e., ILAE (2014 version).

2.It conforms to the diagnostic criteria for refractory epilepsy by the ILAE (2010 version).

3.It conforms to the diagnostic criteria for PFO by the American Society of Echocardiography (ASE) and Society for Cardiac Angiography and Intervention (SCAI) (2015 version). Participants are required to have a second or higher grade of RLS, detected using transthoracic echocardiography with contrast injection, as shown in Figure 4 [2].

4.Patients aged 18 to 55 years-old, who can complete the epilepsy diary independently or with the help of family members.

5.Participants should have at least one epileptic seizure observed during the 6-week recruitment screening period and confirmed by a 24-hour video electroencephalogram (EEG) immediately after the seizure.

6.Participants are required to maintain a stable antiepileptic medical therapy during the screening period and throughout the trial, and to keep their medication regimen as unchanged as possible without an emergency event.

7.Participants were required to have a valid documented epilepsy diary for at least 4 weeks during the recruitment screening period.

8.Participants should agree to participate in this trial and sign an informed consent.

Exclusion criteria

1.Participants with history of pseudo-seizure.

2.Participants with serious mental illness, such as anxiety or depression.

3.Participants with cognitive dysfunction (Mini-Mental State Examination score ≤ 23).

4.Participants with vascular puncture site infection or difficult puncture.

5.Participants with PFO associated with other cardiac structural abnormalities, such as moderate or higher valvular regurgitation and pulmonary hypertension.

6.Participants who have contraindications for antiplatelet therapy such as open trauma fracture surgery, gastrointestinal ulcers, active bleeding, and visceral bleeding three months before the screening period.

7.Participants with existing severe systemic diseases, such as digestive, circulatory, respiratory, liver, urinary, musculoskeletal, immune, or genetic metabolic diseases, whom researchers judge might be a potential impact on the results of the trial.

8.Participants who received head surgery or neuromodulation.

9.Participants under consideration for seizure-related surgery or any surgery involving general anesthesia, or who were already under general anesthesia four weeks before the screening period.

10. Participants who receive vaccinations during the screening period or four weeks before.

11. Participants preparing for pregnancy or breastfeeding during the trial period or three months after the study.

12. Patients who are participating in other interventional clinical studies during the trial.

13.Participants who are planning to travel or live abroad during the study period and cannot be followed-up.

14.Participants deemed inappropriate by the researchers for other reasons (need for detailed recording).

**Table 1. Schedule of enrolment, intervention and assessment according to the Standard Protocol Items: Recommendations for Interventional Trials (SPIRIT) statement**

|  | **Study Period** | | | | | | | | |
| --- | --- | --- | --- | --- | --- | --- | --- | --- | --- |
|  | **Screening** | **Enrolment** | **Allocation** | **Intra-operation** | **Post-operation** | | | | **Close - out** |
| **Time Point*** | **-7 to -2** | **-1** | **-1** | **0** | **4** | **12** | **24** | **48** |  |
|  | | | | | | | | | |
| **Screening** | | | | | | | | | |
| **General information** | **X** |  |  |  |  |  |  |  |  |
| **Eligibility screening** | **X** |  |  |  |  |  |  |  |  |
| **Epilepsy diary** | **X** |  |  |  |  |  |  |  |  |
| **Enrolment** | | | | | | | | | |
| **Informed consent** |  | **X** |  |  |  |  |  |  |  |
| **Allocation** |  |  | **X** |  |  |  |  |  |  |
| **Interventions** | | | | | | | | | |
| **PFO closure** |  |  |  | **X** |  |  |  |  |  |
| **Clopidogrel 75mg** |  |  |  |  | **X** | **X** |  |  |  |
| **Aspirin 100mg** |  |  |  |  | **X** | **X** | **X** |  |  |
| **Assessment** | | | | | | | | | |
| **Seizure frequency** |  |  |  |  | **X** | **X** | **X** | **X** | **X** |
| **Seizure duration** |  |  |  |  | **X** | **X** | **X** | **X** | **X** |
| **Seizure severity** |  |  |  |  | **X** | **X** | **X** | **X** | **X** |
| **Life quality** |  |  |  |  |  | **X** |  | **X** | **X** |
| **Headache score** |  |  |  |  |  | **X** |  | **X** | **X** |
| **Adverse events** |  |  |  |  | **X** | **X** | **X** | **X** | **X** |

**Note: * Times in weeks; PFO, Patent foramen ovale.**

Recruitment of practitioners

The principal investigator will formulate materials containing the information statement and research project protocol consent form. Informed consent will be handed over to each patient and saved strictly. Patients will be recruited according to the predetermined trial recruitment procedure, and all cardiologists and neurologists involved will receive the protocol and training from field workers or through network conferences. Neurologists will be asked to complete an electronic case report form (eCRF) to assess the epilepsy log and relevant risk factors.

To encourage active participation from both participants and researchers, the survey has been designed to be completed in less than 15 minutes. After providing an explanation of the study protocols and goals, all enrolled patients will be required to sign an informed consent form. Researchers and/or research assistants will collect the necessary information, which will be recorded using electronic case report forms (eCRFs) and an online electronic data capture (EDC) system located in the hospital data center. Contributors to the registry will be responsible for data entry and standardization. Once the research is complete and the results are ready to be published, we will share the identified data with others.

**5.3 Informed consent**

Subjects who conform to the inclusion criteria and voluntarily sign informed consent will be included in the study. Subjects are required to sign after carefully reading the informed consent.

A draft version of the informed consent form is provided in supplement draft.

**6. Interventions**

**6.1 Intervention grouping**

Participants in the PFOC-RE trial will be randomly assigned to either the surgical intervention group or the control group. The surgical intervention group will undergo PFO closure surgery and receive antiplatelet therapy for six months. This therapy will include 75mg of clopidogrel and 100mg of aspirin daily for the first three months. From the fourth to the sixth month, the participants will switch to taking 100mg of aspirin daily. Meanwhile, the control group will not receive PFO closure surgery but will still need to undergo antiplatelet therapy for six months with the same medication regimen as the surgical intervention group.

**6.2 Baseline Evaluation**

Baseline protocol-required testing as part of this study are:

- Medical history and risk factors
- Neurology evaluation
- Electrocardiogram (ECG) or Holter study
- Transesophageal echocardiography (TEE) with bubble study
- Magnetic resonance imaging (MRI) or Cat scan (CT)
- Magnetic resonance angiography (MRA), duplex sonography, CT angiography or contrast angiography.

- MRA uses magnetic radio wave energy to look at blood vessels in the body

- Duplex sonography uses high frequency sound waves (Doppler ultrasound) to

measure the size and blood flow through a blood vessel

- CT angiography or contrast angiography uses X-rays and injection of intravenous contrast medium to see the blood flow in the arterial vessel throughout the body

- Pregnancy test (for women of child-bearing potential)
- Blood tests (approximately 4-5 teaspoons or 20-25 ml)

**6.3 Protocol for transesophageal echocardiography (TTE)**

The following recommended protocol for transesophageal echocardiography (TEE) will be used to assure continuity of assessment across study sites. Pre and post-implant echocardiograms should be obtained using image planes in which the atrial septum is perpendicular to the source of ultrasound.

1. Adjusting the instrument

a. Use the highest frequency transducer for patient size.

b. Adjust the transmission power (overall gain) to minimize or eliminate “blooming” of specular reflectors.

c. Adjust the time gain compensation ramp so the image has a uniform brightness to minimize “false positive” dropout in the atrial septum.

d. Place the transmission focus at the depth of the atrial septum.

e. Adjust the color Doppler gain to the point just below the point where random color noise appears.

f. Set the pulse repetition frequency with Nyquist velocity with an approximate range of 35-45 cm/sec.

g. Use a velocity variance map with a “medium” color filter. Set color tissue priority at a level that avoids overlay of color signals onto tissue structures.

h. Perform TEE at 5-7.5 MHz, with all other settings as mentioned above.

i. Perform both longitudinal plane and transverse plane sweeps across the atrial septum.

2. Record 2D Echo in:

a. The Bi-caval longitudinal imaging plane

1. PFO is judged to be present with the visualization of microbubbles in the left atrium within three cardiac cycles from the right atrial opacification at rest and/or during Valsalva release. Evaluate the shunt size by the amount of contrast material that crossed the atrial septum from right to left.

2. The horizontal plane.

3. The aorta from as distally possible to as proximally as possible in the aortic arch.

b. Obtain multiple views of the aortic arch (Omni 90°)

c. Zoom image of left atrial appendage (at least 3 cardiac cycles)

d. Evaluate entire atrial septum including superior region with color flow Doppler in order to exclude sinus venosus defect.

e. Accomplish contrast echocardiography by rapid injection of 5-10ml of isotonic saline solution containing air microbubbles. Perform at least two injections, (1) resting injection and (2) injection with Valsalva maneuver 10 seconds later.

f. Annotate the “Rest” and “Valsalva” injections.

g. Ensure complete opacification of the right atrium. Multiple injections may be required if complete opacification of the right atrium is not obtained. All injections should be labeled as described in 2g.

h. For all digital images ensure that at least 8 cardiac cycles are acquired. Visualization of the initial injection into the right atrium must be included. Ensure annotation of each injection (i.e., “Rest” and “Valsalva”) as per item 2g above.

Note:

TEE will also be used to visualize the presence of an atrial septal aneurysm, which is defined as movement of septum primum greater than or equal to 10mm relative to the plane of the interatrial septum.

An independent Echo Core Lab has been established to review echo tapes of all 6-month TEE videotapes for subjects implanted with a device to evaluate closure of the PFO.

**6.4 Intervention description**

The PFO occluder is a self-expandable, double disc device made from a wire mesh. The two discs are linked together by a short connecting waist. The discs have fabric sewn into them. The fabric used in the device is the same as that used by surgeons to close other cardiac defects. The PFO occluder will remain implanted in patients heart.

Subjects will have the PFO occluder procedure in the cardiac catheterization laboratory, or “cath lab.” Local or general anesthesia will be used and there should not be significant discomfort. This is a non-surgical procedure done using X-ray guidance in which a catheter is inserted through a blood vessel in your thigh up into subjects heart. A catheter is a sterile, flexible, hollow tube that is inserted into a blood vessel to allow for injection or removal of fluids or to pass devices through. After the procedure, subjects may experience some discomfort in the area where the catheter was inserted.

To help doctor evaluate subjects PFO during the procedure, subjects will have a Transesophageal Echocardiography (TEE), angiography and/or maybe intracardiac echocardiography (ICE) done. TEE is an ultrasound test to see subjects’ heart, where an imaging probe is placed through mouth into esophagus (swallowing tube). Some soreness may persist in esophagus for about one week. Angiography is an X-ray of blood vessels or heart chambers filled with a contrast dye that allows doctor to see moving pictures of heart. During angiography, a catheter is passed through a blood vessel in thigh up into heart. If doctor decides to look at heart using ICE, doctors will pass an ICE imaging probe through a blood vessel in thigh up into heart.

After doctor has measured PFO, an PFO occluder is passed through the catheter and positioned in the middle of left atrium. The left disk is deployed, and then drawn against the atrial septum. The right disc is then deployed. After doctor is satisfied with the position of the device, it is released from the delivery cable using a screw mechanism.

The duration of the procedure will be about 1-2 hours. After recovery from anesthesia and a few hours of bed rest, subjects should be able to sit up and walk. Before subjects are discharged from the hospital, a physical exam and electrocardiogram (ECG) will be done to confirm a stable position of the device in your heart. Subjects will be required to take clopidogrel every day for three month and aspirin every day for six months after the procedure.

Doctor may decide to keep you on clopidogrel and aspirin after the required amount of time. To prevent against certain bacterial infections, subjects may need to take some doses of an antibiotic during the first 6 months following the placement of the device, especially if subjects are planning to undergo certain procedures such as dental cleanings. If PFO is not completely closed after 6 months, subjects may continue to receive antibiotics indefinitely for certain procedures.

**7. Randomization and Blinding**

To ensure the equal distribution of patient characteristics between the trial arms, we will use block randomization with variable block length. The R Project for Statistical Computing version 4.2.0 will be used to perform the randomization. Patients will be randomized after completing the enrollment examination and signing informed consent. The allocation pattern will be masked, and the generation and allocation of random numbers will be authorized by a statistician who is not part of the project. The physician who recruits patients will perform the number issuance. Blinding of the surgeon is not feasible due to the pragmatic character of the trial.

Once the subject has completed the baseline examination and has been found to meet all of the Inclusion Criteria and none of the Exclusion Criteria, the site will contact researcher for randomization for each subject. Randomization will occur within 270 days from the date of symptom onset of the index stroke. Subject will be assigned ID numbers at the time of randomization. The subjects randomized to current standard will begin treatment immediately. The subjects randomized to the device group must have their procedure performed within 21 days of randomization. All adverse events occurring after randomization for either study arm will be analyzed according to the statistical plan.

**8. Discontinuation criteria**

1.Serious adverse events occurred during the trail, such as severe cardiovascular and cerebrovascular emergencies, seizures with cardiac arrest, status epilepticus, or other symptoms of serious mental illness.

2.Adverse events occurred during the trail, including allergy to antiplatelet drugs or intolerable side effects, etc.

3.Participants with incomplete data or serious violation of the protocol.

For subjects who are lost to follow-up, attempts to contact the subject will be documented on a Discontinuation CRF. If the subject fails to comply with at least one follow-up visit, the study center must make attempts to contact the subject. There must be at least three attempts to contact the subject, which includes one attempt via registered mail. Details of the attempts must be documented in the subject’s chart.

**9. Adverse Events**

Adverse events are defined as an undesirable health occurrence or untoward deviation in health away from baseline, whether or not device or procedure related.

All adverse events occurring after enrollment (enrollment is defined as point of randomization) for either study group will be collected on the adverse event form and analyzed according to the statistical plan. All adverse events will be continued to be followed until resolution or no resolution is expected.

An independent Data Safety Monitoring Board (DSMB) has been formed to regularly review study progress with regard to safety. Members of the DSMB have no affiliation with the PFO Occluder clinical study. This committee is composed of three clinicians (one Adult Cardiologist, one Neurologist and one Interventional Neuro-radiologist) and a Biostatistician.

The primary responsibilities of the DSMB are to:

1.Establish adverse event definitions (and refine definitions as necessary during the

conduct of the study).

2.Review and adjudicate adverse events as they occur over the course of the study.

3.Review of all failure criteria.

4.Review and validate the patient sample (i.e., inclusion/exclusion deviation and other protocol deviations).

5.Provide oversight for issues affecting general patient welfare.

At any time during the course of the study, the DSMB may offer opinions or make formal recommendations concerning aspects of the study impacting patient safety (e.g., safety related protocol changes or input regarding adverse event rates associated with the Investigational study). Additionally, the DSMB may act as an advisory panel for questions regarding Informed Consent, patient entry, protocol implementation, study endpoints, core lab, Investigator discrepancies, and other issues that may present during the course of the study.

1. Unanticipated Adverse Events: shall be reported by the Investigator to the reviewing IRB as soon as possible, but in no event later than 10 working days after the Investigator first learns of the event.

The Sponsor shall report to all reviewing Institutional Review Boards and participating Investigators, any unanticipated adverse events within 10 working days after the Sponsor first receives notice of the event.

2. Anticipated Adverse Events: adverse events will be classified as either serious adverse events, adverse events or other events and will be identified as either device related, procedure related or “other” (such as pre-existing conditions).

Device failures would include but are not limited to: device removal (due to embolization or misplacement), thrombus formation on the device surface with the risk of subsequent embolization, infectious endocarditis, device collapse due to structural failure, and cardiac perforation due to the presence of the device.

The DSMB met to develop the adverse event definitions. All reported adverse events will be evaluated and categorized as either Serious Adverse Event, Adverse Event, or Other Event based on the following definitions. The DSMB will have the final rule on all adverse events categorizations.

a. Serious Adverse Events

Death, life threatening adverse event, inpatient hospitalization or prolongation of existing hospital stay, persistent or significant disability/incapacity, congenital anomaly/birth defect, and medically significant event, including laboratory abnormalities.

b. Adverse Events

Any reported adverse event not categorized as serious adverse events or other events. Adverse events are defined as an undesirable health occurrence or untoward deviation in health away from baseline, whether or not device or procedure related.

c. Other events

Reported events deemed not to be adverse events by the DSMB. These events may be changes from baseline but not untoward medical occurrences.

The DSMB further defined the following Anticipated Adverse Events. The above mentioned definitions will be applied to the following events to categorize severity. Strong consideration will be given in the determination of procedure or device related for events occurring within 30 days of the procedure. These potential events include, but are not limited to:

Anticipated Adverse Events

Air embolus – symptomatic event resulting from introduction of air into circulatory system

Allergic dye reaction – idiosyncratic reaction to dye used in imaging

Allergic drug reaction – idiosyncratic reaction to drugs used

Anesthesia reactions – idiosyncratic reaction to anesthetic agent used

Apnea – cessation of breathing

Arrhythmia – cardiac rhythm disturbance

Bacterial endocarditis – documented infection of vascular structures including cardiac and related blood vessels

Bleeding – loss of blood from vascular system requiring 1 unit of blood or >5g/dl drop in Hb.

Brachial plexus injury – neurological symptom resulting from damage to brachial

plexus

Cardiac perforation – physical penetration of cardiac structures

Cardiac Tamponade

Chest pain

Device embolization – detachment of a device or its part from intended location

Device erosion

Death

Fever – ≥ 101.5F

Headache/migraine – neurological symptoms attributable to migraine

Hypertension – sustained BP > 140/90mmHg

Hypotension – sustained BP < 90/60mmHg

Myocardial infarction – 2 of the 3 (increased troponin, EKG change, typical chest

pain symptom)

Pacemaker placement secondary to PFO device closure

Palpitations – mild, unsustained

Pericardial effusion – fluid collection around heart documented on 2D-echo

Peripheral embolism – symptom consistent with arterial embolism

Pericarditis – A disorder caused by inflammation of the pericardium, which is the

sac-like covering around the heart

Pleural effusion – excessive fluid in the pleural cavity

Pulmonary embolism – A blockage of an artery in the lungs by fat, air, clumped

tumor cells or a blood clot

Reintervention for device removal

TIA: Acute focal neurological deficit (defined as focal motor deficit, aphasia, difficulty walking, hemisensory deficit, amaurosis fugax, blindness, or focal visual

deficit) presumed due to focal ischemia; symptoms persisting greater than or equal to 5 minutes and less than 24 hours, that is not associated with MR or CT findings of a new, neuroanatomically relevant cerebral infarct.

Thrombus – mass detected on device consistent with thrombus

Valvular regurgitation – Regurgitation of blood from any of the 4 cardiac valves, of more than “mild” severity defined by transthoracic color-flow echo.

Vascular access site injury – damage at vascular access site (i.e. AV fistula, aneurysm)

3. Event Board

A separate Event Board consisting of an Adult Cardiologist, a Neurologist and a Neuroradiologist. The responsibility of the Event Board is to adjudicate events related to primary and secondary neurological endpoint criteria. The Event board will be blinded to the subject’s treatment. The Event Board Committee will have final adjudication responsibilities for subject outcomes related to primary and secondary neurological endpoint criteria.

**10. Outcomes**

**10.1 Primary outcome**

The secondary efficacy outcomes include the percentage decrease in the average duration of seizures after surgery compared with before, improvement in the severity of epilepsy after surgery, frequency of epileptiform discharge by 24h video EEG, evaluation of postoperative quality of life, improvement in migraine in patients with migraine complications, headache impact test, incidence rate of adverse events, cardiac ultrasound index, and some laboratory indicators.

The primary efficacy outcome of our trial is the percentage reduction in the frequency of epileptic seizures during the first year after surgery compared to before surgery. The seizures must conform to the description of seizure characteristics in the International League Against Epilepsy (ILAE), and the preoperative seizure frequency is measured in months and calculated based on valid seizure diaries only. The postoperative seizure frequency is calculated in the same way but should ensure that patients have valid seizure diaries for over 95% of the entire follow-up period.

**10.2 Secondary outcomes**

The secondary efficacy outcomes include the percentage decrease in the average duration of seizures after surgery compared with before, improvement in the severity of epilepsy after surgery, frequency of epileptiform discharge by 24h video EEG, evaluation of postoperative quality of life, improvement in migraine in patients with migraine complications, headache impact test, incidence rate of adverse events, cardiac ultrasound index, and some laboratory indicators.

**10.3 Safety**

Adverse events will be collected from participating subjects. Reported adverse events will be adjudicated by the Data Safety Monitoring Board and analyzed per statistical plan.

Serious adverse events

Death, life threatening adverse event, inpatient hospitalization or prolongation of existing hospital stay, persistent or significant disability/incapacity, congenital anomaly/birth defect, medically significant event, including laboratory abnormalities.

**11. Sample size calculation**

Prior to this study, we recruited 28 subjects with refractory epilepsy to undergo PFO closure surgery and followed them up for one year after the operation. The primary efficacy outcome of this trial is the percentage decrease in the frequency of epileptic seizures during the first year after surgery compared to before surgery. In the 28 patients, the mean percentage decrease was 49.60%, with a standard deviation of 41.67%.

Combined with the control group parameter settings in drug research on refractory epilepsy treatment, we estimate that the surgical group's average therapeutic effect in this study is 50%, with a standard deviation of 0.42. The average percentage decrease in the control group, based on previous literature, is set at 20%, with a standard deviation of 0.20. Therefore, with a superiority margin of 0.1, a single-sided alpha of 0.025, and a test power of 0.80, the total sample size required is 88. To account for possible subject withdrawal and loss of follow-up, we increase the sample size required by 20%. Therefore, we plan to recruit approximately 110 subjects for this study.

12. **Statistical analysis**

1.Program of analysis set

The complete analysis plan consists of three sets: Full Analysis Set (FAS), Per-Protocol Analysis Set (PPS), and Safety Set (SS). Both FAS and SS will include all participants who are randomly assigned, but those with serious protocol violations, active withdrawal, serious adverse events, or extremely poor compliance will be excluded from PPS. For FAS, we intend to follow the modified intention-to-treat (mITT) analysis principles, which will exclude participants without a PFO diagnosis confirmed by intracardiac echocardiography during the surgical procedure.

2.Missing data

In the event that patients are lost to follow-up during the study, their corresponding data will be carried over and included in the analysis. The evaluation process will be performed by the project team's doctors, and the data will be reviewed by the data monitoring committee.

3.Statistical analysis methods

The demographic characteristics of PRE receiving PFO closure and those not receiving it will be compared to assess bias and ensure accurate representation of the participants. Primary and secondary outcomes will be described statistically using mean ± standard deviation, median, minimum, maximum, and other indicators. Categorical data will be described using frequencies and percentages.

The primary outcome of the trial will be analyzed using a mixed model of fixed and random effects, and factors with significant differences between the baseline datasets will be incorporated into the model as control variables. Additional analyses will include t-tests, chi-square tests, rank-sum tests, logistic regression, and propensity score matching to examine the secondary outcome indicators. Covariance analysis and mixed-effects models that consider the effect of time will be used to examine temporal trends in the clinical characteristics of epilepsy. All analyses will be performed using R-project version 4.2.1. Statistical significance will be set at two-sided p<0.05.

**13. Oversight and monitoring**

A. Monitoring Procedures

As indicated by the guidelines established for Investigational Device Exemptions by the NMPA, the following monitoring procedures will be developed for this investigational study. Qualified monitors will be designated to assure that both the Sponsor and Investigators comply with protocol and regulatory requirements. Designee will communicate with each investigational site prior to the onset of the study to review relevant NMPA regulations, the Investigational Plan, IRB review and approval, completion and submission of forms, record keeping requirements, and administrative reports.

During the pre-investigational assessment, designee will assess the adequacy of the facilities, the availability of the Investigator, the potential number of study participants, and the provisions for staff support. To ensure that the Investigators and their staff understand and accept their defined responsibilities, designee will maintain regular correspondence and perform periodic site visits during the course of the study. This will verify the continued acceptability of the facilities, compliance with the Investigational Plan and relevant regulations and guidance documents, and the maintenance of complete records.

Routine monitoring visits will occur during the course of the study as defined in the Clinical Quality Plan. Monitoring will include review and resolution of missing or inconsistent results and source document checks (i.e. comparison of submitted study results to original source documentation) to ensure the accuracy of the reported data.

The monitor will evaluate and summarize the results of each site visit in written reports and a Summary of Visit letter to the Investigator. The Summary of Visit letter will provide documentation of what activities were conducted at the visit as well as any open action items/issues pertaining to the study following each monitoring visit.

Any repeated compliance issues and specific recommendations for resolution of noted deficiencies will be conducted in compliance with our Deviation Management system. The study Clinical Research Associate will promptly review all incoming data to identify inconsistent or missing data and adverse events. Data issues will be identified and addressed to the investigational site for resolution both before and during site visits. To ensure all data are confidential, designee will maintain secure hard copy forms and data files.

B. Data Management

It will be the responsibility of the Principal Investigator to complete and sign all case report forms. Data collected during this study will be analyzed. Additional cases may be performed during the review process and the results submitted to NMPA for review after the completion of the follow-up period previously outlined in this plan.

C. Records and Reports

Evaluation Report Forms

Investigators for this study will be thoroughly instructed in the components and use of the PFO Occluder. Subject case report forms will be distributed to each site before commencing the study. The Investigator, as well as the support staff, will be instructed in the details of the case report forms. A record will be created in a database for each patient in the study. Records will be updated as the study progresses. Additional case report forms will be supplied upon request.

Records are subject to NMPA inspection and must be retained by Investigators for a period of two years after the latter of the following dates:

The date on which the investigation is terminated or completed.

The date the records are no longer required for purposes of supporting an application to the NMPA to market the device.

Investigator Records

Investigators are responsible for maintaining the following records:

Correspondence with the NMPA, the Sponsor, IRB, and other Investigators relating to this investigation.

Record of receipt of the PFO Occluder, and necessary accountability records.

Patient records, including Informed Consent, copies of case report forms, and supporting documents (lab reports, etc.)

Study protocol with dates and reasons for deviations that may affect the scientific quality of the study.

Unanticipated adverse event reports.

Investigator Reports

Investigators are responsible for preparing and submitting the following reports. These reports are subject to NMPA inspection and the retention requirements previously described.

Unanticipated Adverse Effects Related to Device Use

Any occurrence of unanticipated adverse health effect of complications leading to serious injury or death which are associated with the PFO Occluder must be called in immediately (within 24 hours) and reported in writing to the appropriate IRB. This report must be submitted within ten (10) working days after the Investigator first learns of the effect.

Final Report

This report must be submitted within three (3) months after termination or completion of the investigation.

**14. Discussion**

Undeniably, the etiology of refractory epilepsy is complex and difficult to explore, and current drug therapy has not demonstrated satisfactory control efficacy. The PFOC-RE trail will be the first time in the world to preform PFO closure surgery on patients with refractory epilepsy, which may bring encouraging news. This study is hoped to achieve good treatment effects on patients with refractory epilepsy and PFO, including reducing the frequency of postoperative seizures and alleviating the symptoms of seizures.

Reviewing the previous research, the mechanism of PFO's impact on brain neurofunction has gradually been cleared, and there is ample clinical evidence of the health benefits of PFO closure in patients with cryptogenic stroke and migraines[12]. Our clinical trial may further expand the therapeutic value of PFO closure in brain neurofunction diseases. Importantly, our study results can not only provide clinical evidence for exploring the impact mechanism between PFO and refractory epilepsy but also may be used in future larger multicenter clinical trials to benefit more patients.

**15. Trail status**

The first patient was included in December 1, 2022. We expect to finalize the study in November 2026. Protocol version 1.0 document completed in December 2022.

**16. Abbreviations**

Patent Foramen Ovale Closure for Refractory Epilepsy: PFOC-RE; Patent foramen ovale: PFO; Right to left shunt: RLS; Randomized controlled trial: RCT; Electronic data capture: EDC; International League Against Epilepsy: ILAE; Minimum Mental State Examination: MMSE; Electroencephalo-graph: EEG; Full analysis set: FAS; Per-protocol analysis set: PPS; Safety set: SS; Modified intention-to-treat: mITT.

**17. Declarations**

**Ethics approval and consent to participate**

Ethical approval for this study has received from the Ethics Committee of West China Hospital of Sichuan University (No, HXDZ21006), and recruitment started in December 1, 2022. All participants are required to provide written informed consent. Our team confirm that all methods were performed in accordance with the relevant guidelines and regulations. Any protocol amendments will be approved by the appropriate research ethics committee. Study outcomes will be disseminated through peer-reviewed publications and academic conferences.

**18. Appendix**

***DRAFT***

**West China Hospital of Sichuan University**

**The Efficacy and Safety of Patent Foramen Ovale Closure for Refractory Epilepsy (PFOC-RE)**

**A prospectively randomized control trail of an innovative surgical therapy for refractory epilepsy patients with PFO of high-grade right-to-left shunt**

**Standard of Care Treatment**

Patient Consent Form Authorizing Randomization to Treatment with Device under Clinical Investigation, or Current Standard of Care Treatment

Participant's Name:

Date:

**Introduction**

Your doctor has told you that you have had a epilepsy, and you have also been told you have an opening in your heart called a Patent Foramen Ovale (PFO). Normally, the foramen ovale closes at or shortly after birth. In your case, the PFO has remained open and believed to have allowed a blood clot to pass from the right side of your heart to the left side. Blood flowing from the left heart chamber can go to the brain, so a clot or piece of tissue crossing over that opening could go to the brain and cause a refractory epilepsy. This is thought to be the reason that you had a epilepsy and your doctor may have performed a number of tests to confirm this following your epilepsy.

Due to your history of PFO and epilepsy, your doctor has invited you to participate in this research study. This study compares a new product, the PFO occluder device, to current standard of care treatment in preventing another epilepsy or death. The PFO occluder device is implanted in the heart to close a PFO. Current standard of care treatment is medicine to thin your blood. Your doctor has given you this form to tell you about this research study and to ask for your consent to join this research study. Your consent to join the study, if given, will be documented by your signature on the last page of this form, only after you fully understand the study.

**Purpose**

The PFOC-RE trial is a prospective, open-label, randomized, parallel-controlled study designed to investigates the efficacy and safety of PFO closure surgery for the treatment of refractory epilepsy patients, and PFOC-RE followed a 1:1 allocation ratio and was an superiority trail. The primary aim of the trial is to assesses the capacity of PFO closure surgery to alleviate epileptic seizures in patients with epilepsy and PFO. And the PFO occluder device is considered by the Chinese National Medical Products Administration (NMPA) to be an experimental or investigational device.

**Study Procedures**

If you decide to join this research study, you will be randomly assigned to one of two groups: 1) the device group; or 2) the current standard of care treatment group. Being randomly assigned happens by chance, much like the result of flipping a coin. You have an equal chance of being assigned to either group.

**Baseline Evaluation**

You will have the following tests done prior to joining in the study. Some of the testing listed may be part of the testing that you received or may be done specifically for participation in this research project. You should be aware that some tests performed may need to be repeated.

Baseline protocol-required testing as part of this study are:

- Medical history and risk factors
- Neurology evaluation
- Electrocardiogram (ECG) or Holter study
- Transesophageal echocardiography (TEE) with bubble study
- Magnetic resonance imaging (MRI) or Cat scan (CT)
- Magnetic resonance angiography (MRA), duplex sonography, CT angiography or contrast angiography.

- MRA uses magnetic radio wave energy to look at blood vessels in the body

- Duplex sonography uses high frequency sound waves (Doppler ultrasound) to

measure the size and blood flow through a blood vessel

- CT angiography or contrast angiography uses X-rays and injection of intravenous contrast medium to see the blood flow in the arterial vessel throughout the body

- Pregnancy test (for women of child-bearing potential)
- Blood tests (approximately 4-5 teaspoons or 20-25 ml)

**Current Standard of Care Treatment Group**

If you are randomly assigned to the current standard of care treatment group, you will receive medicine to thin your blood, so that the potential chance if having seizure is decreased and the benefit of prevent stroke also is existed. This therapy includes 75mg of clopidogrel and 100mg of aspirin daily for the first three months. From the fourth to the sixth month, the participants switch to taking 100mg of aspirin daily.

**Device Group**

If you are randomly assigned to the device group, you will have the PFO occluder implanted in your heart to close your PFO. You will be required to take clopidogrel every day for one month and aspirin every day for six months after the procedure which may be continued for a longer period of time at the discretion of your physician.

The PFO occluder is a self-expandable, double disc device made from a wire mesh. The two discs are linked together by a short connecting waist. The discs have fabric sewn into them. The fabric used in the device is the same as that used by surgeons to close other cardiac defects. The PFO occluder will remain implanted in your heart.

You will have the PFO occluder procedure in the cardiac catheterization laboratory, or “cath lab.” Local or general anesthesia will be used and there should not be significant discomfort. This is a non-surgical procedure done using X-ray guidance in which a catheter is inserted through a blood vessel in your thigh up into your heart. A catheter is a sterile, flexible, hollow tube that is inserted into a blood vessel to allow for injection or removal of fluids or to pass devices through. After the procedure, you may experience some discomfort in the area where the catheter was inserted.

To help your doctor evaluate your PFO during the procedure, you will have a Transesophageal Echocardiography (TEE), angiography and/or maybe intracardiac echocardiography (ICE) done. TEE is an ultrasound test to see your heart, where an imaging probe is placed through your mouth into your esophagus (your swallowing tube). Some soreness may persist in your esophagus for about one week. Angiography is an X-ray of blood vessels or heart chambers filled with a contrast dye that allows your doctor to see moving pictures of your heart. During angiography, a catheter is passed through a blood vessel in your thigh up into your heart. If your doctor decides to look at your heart using ICE, he will pass an ICE imaging probe through a blood vessel in your thigh up into your heart.

After your doctor has measured your PFO, an PFO occluder is passed through the catheter and positioned in the middle of your left atrium. The left disk is deployed, and then drawn against the atrial septum. The right disc is then deployed. After your doctor is satisfied with the position of the device, it is released from the delivery cable using a screw mechanism.

The duration of the procedure will be about 1-2 hours. After recovery from anesthesia and a few hours of bed rest, you should be able to sit up and walk. Before you are discharged from the hospital, a physical exam and electrocardiogram (ECG) will be done to confirm a stable position of the device in your heart. You will be required to take clopidogrel every day for one month and aspirin every day for six months after the procedure.

Your doctor may decide to keep you on clopidogrel and aspirin after the required amount of time. To prevent against certain bacterial infections, you may need to take some doses of an antibiotic during the first 6 months following the placement of the device, especially if you are planning to undergo certain procedures such as dental cleanings. If your PFO is not completely closed after 6 months, you may continue to receive antibiotics indefinitely for certain procedures.

**Follow-up Visits**

The study requires a follow-up exam when you leave the hospital (device group patients only), and follow-up visits at 1 month, 3 month, 6 months, 1 year, and annually after that until the completion of the study. Follow-up visits may include a physical, neurological exam, ECG or Holter monitor, and a TEE (device group only) at 6 months. Beginning at the 1 year follow-up visit, your physician may conduct the follow-up visits by telephone.

If during that telephone visit, it is determined that you may have had a medical problem, you may be asked to come for an in-person follow-up visit. During each of these visits you will be asked about your health since your previous visit. You will be asked a series of questions about whether you have had any seizure during this period. If it is determined that you had seizure, 30 days after your symptoms of the seizure, you will undergo an additional neurologic exam and blood tests (approximately 5-6 teaspoons or 25-30 ml), and a neurologist will determine the severity of the seizure. You will be followed until the device is NMPA approved or until the study is complete. It is important to keep all follow-up appointments that are scheduled for you.

**Potential Benefits**

A potential benefit of having the PFO occluder implanted is that your PFO may be closed without open-heart surgery. Usually the pain after the implant procedure is much less than open-heart surgery. Your stay in the hospital is usually much shorter. The recovery time is much faster and there is little scarring. However, it is not known how well the PFO occluder will work in humans and is to be evaluated in this study.

The potential benefits of having the current standard of care treatment are that your condition will be treated without any invasive procedures, you will be closely followed, and data from this study may change future treatments for refractory epilepsy patients.

**Potential Risks**

Potential adverse events specific to device placement include, but are not limited to:

- Air embolus-leakage of air into the veins or heart
- Allergic reaction to contrast dye or medications used in the study
- Apnea – (a condition where your breathing slows down or stops)
- Bacterial endocarditis - infection in the heart or blood vessels
- Bleeding
- Brachial plexus injury -injury to a group of nerves around your shoulder
- Cardiac Arrhythmia (abnormal heart beats)
- Cardiac perforation-perforation of the heart
- Cardiac Tamponade-large amount of fluid collection around your heart
- Chest Pain
- Headache/migraine
- Device embolization-movement of device from its intended site
- Device erosion-device wear on nearby tissues or cardiac structures
- Death
- Fever
- Hypertension – (high blood pressure)
- Hypotension - (low blood pressure)
- Myocardial Infarction-heart attack
- Pacemaker placement secondary to PFO device closure
- Palpitations-feeling of pounding or fluttering in the chest
- Pericardial effusion-Fluid collection around your heart
- Peripheral embolism- an obstruction in a blood vessel of the body other than the heart and brain due to a blood clot or other foreign matter that gets stuck while traveling through the bloodstream
- Pulmonary embolism – A blockage of an artery in the lungs by fat, air, clumped tumor cells, or a blood clot
- Pericarditis- A disorder caused by inflammation of the pericardium, which is the sac-like covering around the heart
- Pleural effusion-fluid collection around your lungs
- Re-Intervention for device removal
- Thrombus-formation of blood clots on the device
- Valvular regurgitation-backflow of blood through your heart valves
- Vascular access site injury- bleeding, discomfort and/or bruising around the place where the catheter is inserted into the thigh

Procedural complications related to PFO closure with a device include but is not limited to:

- allergy to the contrast dye
- bleeding around introducer sheaths (a small plastic tube which is used as access for catheters into an artery/vein during an angiogram procedure)
- leakage of air into the veins or heart
- cardiac arrhythmia (abnormal heart beats)
- Infection
- injury to the artery, vein or nerves in the groin or neck needing surgical repair
- tear or puncture of the heart (during the procedure) or esophagus (from the use of the TEE probe).

There are also risks associated with the use of anesthesia. Subjects may experience a reaction to the anesthesia medication. Additionally, subjects allergic to nickel may suffer an allergic reaction to this device.

If during the device implant procedure, the device were to be dislodged, you would need surgery for its removal and at the same time the heart defect would be repaired. Cardiac surgery following device placement may be more difficult. Since this device is investigational, the potential risks to the embryo and fetus are unknown in females who may become pregnant during the course of the study. Because the PFO occluder device is new there may be other risks that are not known at this time.

Reported risks associated with medicine used to thin the blood include, but are not limited to, increased bleeding time, GI bleeding, drowsiness, dizziness, headache, nausea and vomiting, rash, and bruising. There may be other risks related to medicine used to thin the blood that are unknown at this time.

**Alternative Treatment**

If you choose not to participate in this study at this time, your doctor will discuss alternative treatments with you. Treatment options may include open-heart surgery to repair your PFO, the use of drugs other than those planned for this study to prevent the occurrence of refractory epilepsy, or not having any treatment at all.

**Confidentiality**

Any information obtained from this study that can be identified with you will remain confidential and will be disclosed only with your permission. In any written reports or publications, you will not be identified or identifiable and only combined data will be presented. By signing this consent you are authorizing access to your medical records, which may be made available to the NMPA.

**Payments and Cost**

(Option 1: Device and implant procedure being paid for by West China Hospital of Sichuan University)

You will not receive any payments for participating in this study. For this research study, the sponsor (Chinese Academy of Medical Sciences) has agreed to pay for the costs related to the study device and the procedure to implant the device. All testing and services performed solely for this study will also be provided to you at no cost. You are responsible for all other costs (including co-pays and deductibles) that are part of your usual medical care and that would have been incurred regardless of your enrollment in the study. If you have any questions about your health insurance, or possible expenses, please talk with your doctor and your health insurer or Medicare.

(Option 2: Device and implant procedure being submitted to insurance/third party payer coverage)

You will not receive any payments for your participation in this study. You or your third party payer must pay for the implant procedure and all tests that are done in conjunction with this procedure. In the event that this research study causes any injury, treatment for that injury including surgery, first aid, and emergency care will be available as needed. You or your third party payer must provide payment for any such treatment, which is similar to the treatment that you would receive if an injury occurred during the standard treatment for your condition. You should not incur any additional expenses for participating in this clinical study.

**Whom to Contact**

During the course of this clinical trial, if you have any further questions about this study or your rights as a research subject, concerns, or research-related injuries connected to your participation, please contact the Investigator below.

Investigator:

Phone Number:

**AUTHORIZATION TO USE AND DISCLOSE**

**PROTECTED HEALTH INFORMATION**

I agree to permit the investigator and staff members, the sponsor of the PFOC-RE Clinical Trial, to use and disclose health information that identifies me for the purposes described below. I also agree to permit {West China Hospital of Sichuan University}, my doctors, and my other health care providers to disclose health information in my medical records to the Researchers, and the NMPA for the purposes described below.

1. The health information that may be used and disclosed includes:

 All information collected during the research as described in the Informed Consent Form; and Health information in my medical records that is relevant to the research described in the Informed Consent Form.

2. The Researchers may:

 Use and share my health information to conduct the research;

 Disclose my health information to the sponsor of research;

 Disclose my health information as required by law;

 Disclose my health information to representatives of government organizations and other persons who are required to watch over the safety and effectiveness of medical products and therapies and the conduct of research; and Remove from my health information my name and other information that could be used to identify me.

4. Once information that could be used to identify me has been removed, the information that remains is no longer subject to this Authorization and may be used and disclosed by the Researchers as permitted by law.

5. Once my health information has been disclosed to a third party, laws may no longer protect it from further disclosure. However, the Researchers agree to protect my health information by using and disclosing it only as permitted by me in this Authorization and the Informed Consent. Also, no publication about the research will reveal my identity without my specific written permission. These limitations continue even if I revoke (take back) this Authorization.

6. Please note that:

 You do not have to sign this Authorization, but if you do not, you will not be allowed to participate in the research.

 You may change your mind and revoke this authorization at any time. To revoke this Authorization, you must write to {name and contact information}. However, if you revoke this Authorization, you will no longer be allowed to participate in the research. Also, even if you revoke this Authorization, the information already obtained by the Researchers may be used and disclosed as permitted by the Authorization and the Informed Consent.

 While the research is in progress, you will not be allowed to see your health information that is created or collected in the course of the research. After the research is finished, however, you may see this information as described in the Notice of Information practices.

7. This Authorization does not have an expiration (ending) date.

8. You will be given a copy of this Authorization after you have signed it.

Print Name of Subject

Date

Signature of Subject/Legally Authorized Representative

Date

Signature of Witness (if required)
