# Supplementary material for: The efficacy and safety of patent Foramen Ovale Closure for Refractory Epilepsy (PFOC-RE): a prospectively randomized control trial of an innovative surgical therapy for refractory epilepsy patients with PFO of high-grade right-to-left shunt
Source: BMC Neurol. 2023 Jul 27;23:282. doi: 10.1186/s12883-023-03317-0 (PMC10373383; doi:10.1186/s12883-023-03317-0)
Supplement: Supplementary file 3 — Supplementary Material 3 [file 12883_2023_3317_MOESM3_ESM.docx]

**Supplement File 2. PFOC-RE Trail Registration Data**

| **Data category** | **Information** |
| --- | --- |
| **Primary registry and trial identifying number** | **ClinicalTrials.gov, ChiCTR2200065681** |
| **Date of registration in primary registry** | **11 November, 2022** |
| **Secondary identifying numbers** | **NA** |
| **Source(s) of monetary or material support** | **Medical and Health Technology Innovation Project of Chinese Academy of Medical Sciences** |
| **Primary sponsor** | **Medical and Health Technology Innovation Project of Chinese Academy of Medical Sciences** |
| **Secondary sponsor(s)** | **1-3-5 project for disciplines of Excellence Clinical Research Incubation Project** |
| **Contact for public queries** | **LC, MD, [[leilei_25@126.com](mailto:[leilei_25@126.com)]** |
| **Public title** | **The Efficacy of** **Patent Foramen Ovale Closure for Refractory Epilepsy in Adults: A Randomized Controlled Trial** |
| **Scientific title** | **The Efficacy of Patent Foramen Ovale Closure for Refractory Epilepsy in Adults: A Randomized Controlled Trial** |
| **Countries of recruitment** | **China** |
| **Health condition(s) or problem(s) studied** | **Patent Foramen Ovale Closure, *Refractory Epilepsy*** |
| **Intervention(s)** | **Active comparator: Patent Foramen Ovale Closure plus 6 months of antiplatelet therapy** |
|  | **Placebo comparator: 6 months of antiplatelet therapy** |
| **Key inclusion and exclusion criteria** | **Ages eligible for study: Between 18 years and 55 years  Sexes eligible for study: Both Accepts healthy volunteers: No** |
|  | **Inclusion criteria:** **See supplement file 3** |
|  | **Exclusion criteria: See supplement file 3** |
| **Study type** | **Interventional** |
|  | **Allocation: Randomized, parallel assignment intervention model with open label** |
|  | **Primary purpose: Treatment** |
|  | **Phase III** |
| **Date of first enrolment** | **December 2022** |
| **Target sample size** | **110** |
| **Recruitment status** | **Recruiting** |
| **Primary outcome(s)** | **The percentage decrease in the frequency of epileptic seizures during the first year after surgery compared with that before surgery** |
| **Key secondary outcomes** | **The percentage decrease in the average duration of seizures after operation compared with before**  **The severity of epilepsy improved after surgery**  **The frequency of epileptiform discharge by 24h video EEG** |
