# Supplementary material for: The efficacy and safety of patent Foramen Ovale Closure for Refractory Epilepsy (PFOC-RE): a prospectively randomized control trial of an innovative surgical therapy for refractory epilepsy patients with PFO of high-grade right-to-left shunt
Source: BMC Neurol. 2023 Jul 27;23:282. doi: 10.1186/s12883-023-03317-0 (PMC10373383; doi:10.1186/s12883-023-03317-0)
Supplement: Supplementary file 4 — Supplementary Material 4 [file 12883_2023_3317_MOESM4_ESM.docx]

**Supplement File 3. Objectives, Endpoints, and Criteria for inclusion and exclusion of patients of PFOC-RE**

| **Objective** | **Endpoint** |
| --- | --- |
| **Primary** |  |
| - **To evaluate the efficacy of** **Patent Foramen Ovale Closure surgery in the treatment of refractory epilepsy patients by the decrease in the frequency of seizures during the postoperative follow-up period** | - **The percentage decrease in the average monthly frequency of epileptic seizures during the first year after surgery compared with that before surgery** |
| **Major Secondary** |  |
| - **To evaluate the improvement effect of Patent Foramen Ovale Closure on the duration of seizure in patients with refractory epilepsy** | - **The percentage decrease in the average duration of seizures after operation compared with before** |
| **Other Secondary** |  |
| - **To evaluate the improvement effect of** **Patent Foramen Ovale Closure on other seizure characteristics in patients with refractory epilepsy** | - **The severity of epilepsy improved after surgery** - **The frequency of epileptiform discharge by 24h video EEG** - **Postoperative quality of life evaluation** - **Migraine improvement in patients with migraine complications** |
| - **To evaluate the safety of the Patent Foramen Ovale Closure** | - **Headache impact test** - **Incidence rate of adverse events** - **Cardiac ultrasound index after surgery** |
| **Inclusion criteria**   - **It conforms to the diagnostic criteria for epilepsy by the International League Against Epilepsy, i.e., ILAE (2014 version).** - **It conforms to the diagnostic criteria for refractory epilepsy by the ILAE (2010 version).** - **It conforms to the diagnostic criteria for PFO by the American Society of Echocardiography (ASE) and Society for Cardiac Angiography and Intervention (SCAI) (2015 version). Participants are required to have a second or higher grade of RLS, detected using transthoracic echocardiography with contrast injection, as shown in Figure 4 [2].** - **Patients aged 18 to 55 years-old, who can complete the epilepsy diary independently or with the help of family members.** - **Participants should have at least one epileptic seizure observed during the 6-week recruitment screening period and confirmed by a 24-hour video electroencephalogram (EEG) immediately after the seizure.** - **Participants are required to maintain a stable antiepileptic medical therapy during the screening period and throughout the trial, and to keep their medication regimen as unchanged as possible without an emergency event.** - **Participants were required to have a valid documented epilepsy diary for at least 4 weeks during the recruitment screening period.** - **Participants should agree to participate in this trial and sign an informed consent.** | |
| **Exclusion criteria**   - **Participants with history of pseudo-seizure.** - **Participants with serious mental illness, such as anxiety or depression.** - **Participants with cognitive dysfunction (Mini-Mental State Examination score ≤ 23).** - **Participants with vascular puncture site infection or difficult puncture.** - **Participants with PFO associated with other cardiac structural abnormalities, such as moderate or higher valvular regurgitation and pulmonary hypertension.** - **Participants who have contraindications for antiplatelet therapy such as open trauma fracture surgery, gastrointestinal ulcers, active bleeding, and visceral bleeding three months before the screening period.** - **Participants with existing severe systemic diseases, such as digestive, circulatory, respiratory, liver, urinary, musculoskeletal, immune, or genetic metabolic diseases, whom researchers judge might be a potential impact on the results of the trial.** - **Participants who received head surgery or neuromodulation.** - **Participants under consideration for seizure-related surgery or any surgery involving general anesthesia, or who were already under general anesthesia four weeks before the screening period.** - **Participants who receive vaccinations during the screening period or four weeks before.** - **Participants preparing for pregnancy or breastfeeding during the trial period or three months after the study.** - **Patients who are participating in other interventional clinical studies during the trial.** - **Participants who are planning to travel or live abroad during the study period and cannot be followed-up.** - **Participants deemed inappropriate by the researchers for other reasons (need for detailed recording).** | |
